# SliceMap: a binary classification-driven 2D pipeline for detecting discriminative candidate regions in brain MRI

**DOI:** 10.3389/fnimg.2026.1819355

**Published:** 2026-05-15

**Authors:** Xiaoye Jiang, Zhijin Wu, Zhaohui S. Qin

**Affiliations:** 1 Department of Biostatistics and Bioinformatics, Emory University, Atlanta, GA, United States; 2 Department of Biostatistics, Brown University, Providence, RI, United States

**Keywords:** binary classification modeling, brain MRI, convolutional neural network, occlusion-based attribution analysis, sex difference

## Abstract

Identifying spatially localized neuroanatomical signals from brain magnetic resonance imaging (MRI) is central to many clinical and scientific questions, including medical diagnosis and the investigation of disease mechanisms. However, reliably detecting such subtle and spatially localized signals from high-dimensional MRI data remains challenging. In our study, we propose a performance-guided two-dimensional (2D) slice-based pipeline for identifying candidate spatial regions in brain MRI. The pipeline employs efficient 2D convolutional neural networks trained on plane-specific MRI slices, using binary classification as a modeling task to evaluate slice-level performance. The best-performing slice from each anatomical plane is then subjected to occlusion-based attribution analysis, and the resulting maps are jointly examined to localize a candidate three-dimensional brain region. We demonstrate the proposed pipeline using sex classification as a controlled testbed, identifying a spatially localized candidate region corresponding to the anterior cingulate cortex and adjacent medial structures including portions of the corpus callosum genu, consistent with prior neuroanatomical findings on sex differences in these regions.

## 1 Introduction

Brain magnetic resonance imaging (MRI) plays a central role in neuroscience and clinical research by enabling the noninvasive study of brain structure (Matthews and Jezzard, 2004; Pinter and Fritz, 2020). A central goal of many MRI-based analyses is to identify spatially localized neuroanatomical regions that distinguish disease-associated effects or phenotypic contrasts (Ashburner and Friston, 2000; Frisoni et al., 2010; Good et al., 2001). Such spatial localization is crucial for medical diagnosis (Frisoni et al., 2010; Freedman, 2006; Teipel et al., 2013), for advancing our understanding of the pathological and physiological mechanisms underlying these differences (Dijkhuizen, 2006; Dijkhuizen and Nicolay, 2003), and for guiding hypothesis generation in downstream biological interpretation (Woo et al., 2017).

However, reliably identifying such spatially localized signals from brain MRI remains challenging. MRI data are high-dimensional and spatially complex, and many clinically relevant signals are subtle and not readily apparent to the human eye (Tran et al., 2013).

Consequently, analytical approaches, including statistical modeling (Aja-Fernández and Vegas-Sánchez-Ferrero, 2016) and machine learning methods (Mohsen et al., 2012), have been developed over time to address these challenges. Traditional approaches, such as voxel-based morphometry (VBM), typically perform voxel-wise analyses or rely on linear modeling assumptions, which limits their ability to capture nonlinear patterns and to model coordinated effects across brain regions (Ashburner and Friston, 2000). With the development of machine learning techniques, nonlinear patterns can be effectively captured. However, standard machine learning methods (e.g., Support Vector Machine, Random Forest, Elastic Net, Kernel Ridge Regression) require pre-defined feature engineering, which introduces prior assumptions about task-relevant brain features, thereby limiting the ability to detect novel and meaningful patterns in MRI images (Abrol et al., 2021).

More recently, deep learning techniques address these limitations by enabling automated representation learning directly from raw image data, while preserving the spatial structure of MRI images and capturing complex, distributed patterns across brain regions (Smucny et al., 2022). Among deep neural network architectures, convolutional neural networks (CNNs) have demonstrated strong performance in structural MRI analytical tasks, offering a practical balance between representational capacity and computational feasibility in neuroimaging cohorts (Khagi and Kwon, 2020; Lu et al., 2022; Farooq et al., 2017). Specifically, CNNs learn features at multiple spatial scales through successive convolutional layers, from local edge and texture patterns to higher-level structural configurations, making them well-suited for structural MRI where pathological changes manifest across a range of spatial extents (Dong et al., 2025). Furthermore, parameter sharing across spatial locations reduces model complexity, enabling effective training on moderately sized neuroimaging cohorts without requiring large-scale computational resources (Lecun et al., 1998). Prior work (e.g., Yu et al., 2024) has further demonstrated robust CNN-based performance on structural MRI datasets.

In modern MRI analysis pipelines, deep learning models are typically trained to discriminate phenotypic classes, and regions of interest are subsequently inferred through model-based attribution techniques, such as gradient-based attribution and occlusion strategies (Zeiler and Fergus, 2014; Zhang et al., 2021; Korolev et al., 2017; Shojaei et al., 2023). Gradient-based methods, such as GradCAM (Selvaraju et al., 2017), generate saliency maps by leveraging class-specific gradient information from convolutional layers, but their spatial resolution is constrained by the feature map dimensionality of the final layer. Perturbation-based methods, such as occlusion (Zeiler and Fergus, 2014), instead directly measure changes in model output upon systematic masking of input regions, offering more direct spatial interpretability.

Taken together, these considerations motivate the use of CNN-based predictive modeling combined with occlusion-based attribution for spatial localization in MRI data.

However, existing deep learning-based spatial localization pipelines predominantly operate on full three-dimensional brain volumes, imposing substantial computational demands and typically requiring large training cohorts. Moreover, such pipelines are often developed for specific phenotypic contrasts and are not explicitly designed as reusable frameworks generalizable across different phenotypic classification tasks (Zhang et al., 2015, 2020; Hosseini-Asl et al., 2016).

To address these limitations, we develop SliceMap, a framework that first evaluates slice-wise classification performance across anatomical planes to identify the most discriminative slices and subsequently applies occlusion-based attribution within these slices to localize candidate regions. It leverages a slice-based two-dimensional pipeline to identify three-dimensional discriminative brain regions in MRI. This design provides (1) an efficient strategy for three-dimensional spatial localization without requiring full volumetric modeling, and (2) a framework designed to be applicable across different phenotypic contrasts, demonstrated here on sex classification as a proof of concept.

## 2 Materials and methods

**Figure 1:**
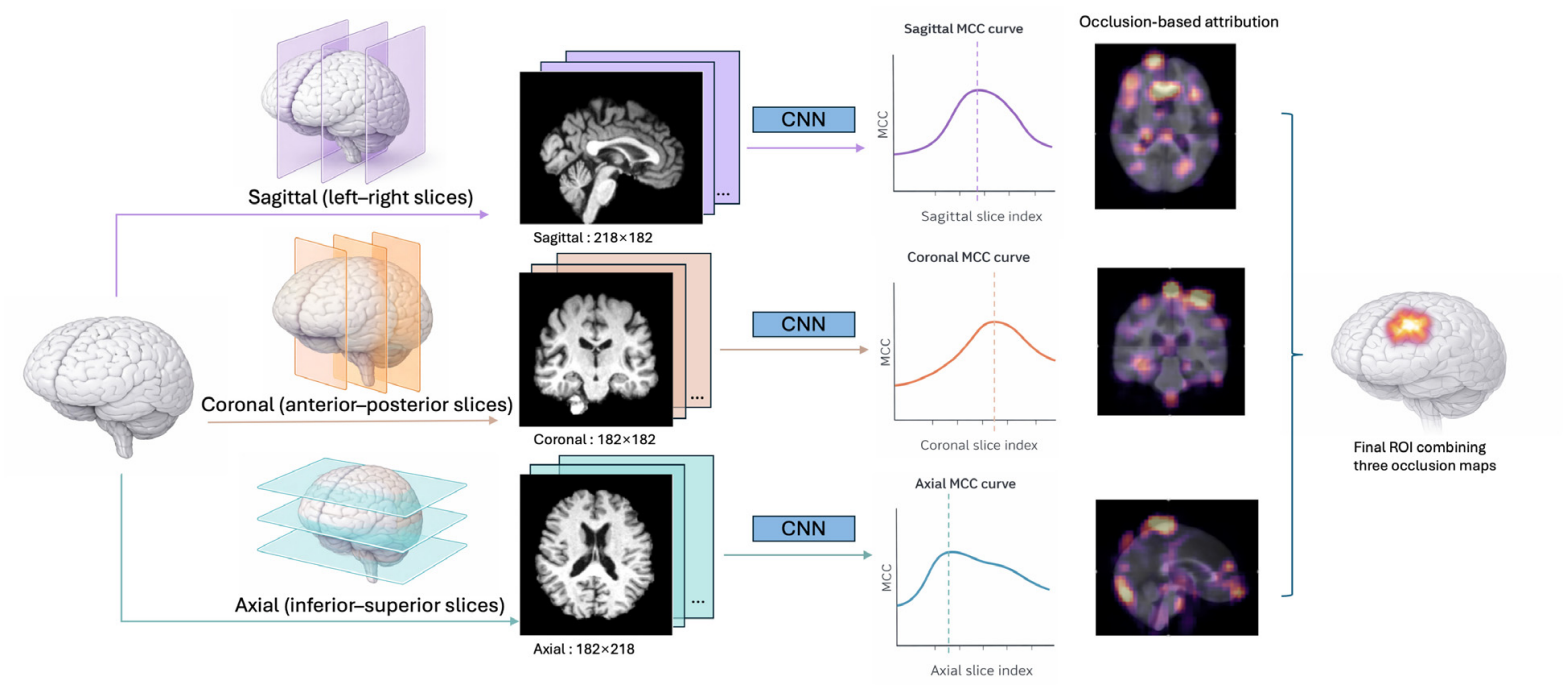
Overview of the proposed SliceMap pipeline for spatial localization of discriminative neuroanatomical signals. The schematic illustrates the core analytical workflow: 2D slices are extracted from 3D brain MRI volumes along the sagittal, coronal, and axial planes, and slice-wise classification performance is evaluated independently within each plane using a 2D CNN. The best-performing slice from each plane is identified based on MCC. Occlusion-based attribution analysis is then applied to each best-performing slice to generate spatial attribution maps. The high-attribution regions across the three planes are jointly examined to identify a candidate three-dimensional ROI associated with the modeled binary contrast.

The overall workflow of the proposed SliceMap pipeline is illustrated in Figure 1. Sex classification is used as a test case to demonstrate and validate the pipeline.

### 2.1 Data preparation

T1-weighted structural MRI scans were originally obtained from the Alzheimer's Disease Neuroimaging Initiative (ADNI) (Jack et al., 2008), including data from ADNI1, ADNI-GO/2, and ADNI3. For the present study, we used preprocessed structural MRI image data generated according to the procedure described in a previous study (Yu et al., 2024). Building upon these data, we performed additional intensity normalization using MONAI (Cardoso et al., 2022) to standardize voxel intensity distributions across samples before modeling.

After preprocessing, 9,938 MRI scans (5,379 males, 4,559 females) from 2,258 participants were retained. Demographic characteristics, including age and diagnostic group composition, are summarized in Supplementary Table S1.

To prevent data leakage, data splitting was performed at the subject level rather than the image level. Subjects were randomly divided into training, validation, and testing sets in a 64:16:20 ratio, corresponding to 1,444, 362, and 452 subjects, respectively, and yielding 6,334 training, 1,568 validation, and 2,036 testing images.

Because our framework operates on two-dimensional inputs, we extracted slice-wise representations from the three orthogonal anatomical planes—sagittal, coronal, and axial—corresponding to left–right, front–back, and superior–inferior orientations of the brain (Toga and Thompson, 2001). This slice-wise representation forms the basis for all subsequent classification and analysis steps and enables the identification of anatomically localized sex-linked signals.

### 2.2 Methods

This section describes the methodological framework used in this study, including the construction and training of the CNN model for classification, as well as a set of statistical validation and spatial localization analyses. Specifically, these analyses include simulation-based sensitivity testing and permutation-based specificity testing to assess signal validity, dual-threshold analysis to examine how classification performance and sample retention change with varying prediction confidence thresholds, and localization procedures to identify anatomically localized sex-linked patterns in brain MRI.

#### 2.2.1 CNN architecture

The CNN architecture was adapted from the 2D convolutional neural network, as described in an earlier study (Yu et al., 2024), which demonstrated strong performance for MRI-based sex classification task. Also, batch normalization layers were applied after each convolutional layer to stabilize training and facilitate convergence (Bjorck et al., 2018; Santurkar et al., 2018).

The final network comprised three convolutional blocks, each consisting of a 3 × 3 convolutional layer, Batch Normalization, a rectified linear unit (ReLU), and a 2 × 2 max-pooling layer with stride 2. The number of feature maps increased progressively from eight to 16 to 32 across the three blocks. Following the final pooling layer, feature maps were flattened and passed through a fully connected layer with 128 hidden units. Layer normalization, ReLU activation, and dropout (rate = 0.5) were then applied, followed by a final linear layer producing a single logit for binary classification.

#### 2.2.2 Model training and hyperparameter optimization

For each anatomical plane, slice-specific models were trained independently. All models were implemented in PyTorch (Meta Platforms, Inc., Menlo Park, CA, United States, v2.5.1 with CUDA 12.4) and trained on a single NVIDIA A30 GPU (24 GB memory). Training was performed using the AdamW optimizer (Loshchilov and Hutter, 2019) with an initial learning rate of 5 × 10^−5^ and a batch size of 64, and binary cross-entropy with logits loss (BCEWithLogitsLoss) for up to 200 epochs. Model checkpoints were saved after each epoch, and the checkpoint achieving the lowest validation loss was selected for evaluation on the independent test set.

Hyperparameter optimization was conducted using the sagittal mid-slice CNN model. The following hyperparameters were explored: weight decay {0.1, 0.01, 0.001}; learning-rate scheduling using StepLR with step sizes of 5, 10, or 15 epochs (decay factor γ = 0.5), as well as a fixed learning-rate setting without scheduling; and the inclusion or exclusion of batch normalization layers. Each configuration was trained for 200 epochs using identical data splits, optimizer, and loss function, and repeated ten times with different random initializations. The mean Matthews correlation coefficient (MCC) across repetitions was used to select the final configuration, which was then fixed and applied to all slice-specific models within each anatomical plane.

#### 2.2.3 Evaluation metric

Although the sex distribution in the dataset was approximately balanced (male: 5,379; female: 4,559), the proposed pipeline is intended to be applicable to a broad range of binary classification tasks in neuroimaging, many of which may exhibit substantial class imbalance. Therefore, MCC was adopted as the primary evaluation metric, as it provides a symmetric and robust measure of binary classification performance and is less sensitive to class imbalance (Chicco and Jurman, 2020). The MCC is defined as


$$\begin{array}{l} MCC=\frac{TP\times TN-FP\times FN}{\sqrt{\left(TP+FP\right)\left(TP+FN\right)\left(TN+FP\right)\left(TN+FN\right)}} \end{array}$$ (1)

Notably, in binary classification, MCC is mathematically equivalent to the Pearson correlation coefficient computed between the observed and predicted binary labels. We also reported accuracy and precision to provide a more complete characterization of model performance.

#### 2.2.4 Simulation experiment

To evaluate whether the CNN can reliably identify spatially localized discriminative information, we conducted a controlled simulation experiment. Specifically, a predefined cubic region ([25:34]^3^) was selected from the original dataset. Within this region, voxel intensities in female MRI volumes were modified by replacing the original values with samples drawn from a Gaussian distribution whose mean was set to twice the original intensity and whose standard deviation was fixed at one. All voxels outside this region remained unchanged, and male MRI volumes were left unmodified.

Both the original and simulated datasets were analyzed using identical training and evaluation procedures. For each anatomical plane (sagittal, coronal, and axial), slice-wise CNN models were applied to slices within the index range of 5–65, encompassing both slices intersecting the simulated cubic region and adjacent unaffected slices. Model performance was quantified using MCC, enabling direct slice-wise comparisons between the original and simulated datasets. Because the simulated data differed from the original data only through the injected signal within the predefined cubic region, this experimental design provides a controlled means to assess whether the model can localize and exploit discriminative signals at a known spatial location.

#### 2.2.5 Permutation study

To verify that CNN learned genuine structural information rather than random noise, a permutation experiment was performed. Identity labels were randomly shuffled across subjects while keeping all images and training settings identical to the original configuration. For each anatomical plane, the model was retrained using the permuted labels, and its performance was compared with that of the model trained under the true labels.

The significance of the performance difference between the two conditions was assessed using a two-sample *t*-test across 10 repeated runs. A consistently higher MCC under the true-label condition would confirm that CNN captured biologically meaningful sex-linked patterns rather than spurious correlations.

#### 2.2.6 Dual-threshold analysis

To evaluate how model performance varies with prediction confidence, we applied a dual-threshold strategy to the probabilistic outputs. In this framework, predictions with probabilities above an upper threshold were classified as male, those below a lower threshold as female, and intermediate predictions were ignored.

For each threshold, MCC was computed based on the retained samples, and the proportion of retained samples was recorded.

This analysis quantifies how model performance changes as the decision criterion becomes more stringent, revealing the trade-off between classification performance and the fraction of samples confidently classified by the model.

#### 2.2.7 Spatial localization analysis

To detect discriminative candidate regions, we implemented a two-step spatial analysis procedure.

First, to identify anatomical slices carrying stronger sex-discriminative information, we performed a slice-wise analysis across the sagittal, coronal, and axial planes. For each plane, approximately 40 slices were selected to span the entire brain volume, with denser sampling near the mid-slice region where structural detail is typically richer (Toga and Thompson, 2001). Slice-wise discriminative performance was primarily quantified using the MCC.

Second, to spatially localize candidate discriminative regions within each plane, we applied occlusion-based attribution analysis to the best-performing slice identified in each anatomical plane. For each slice, an occlusion heatmap was generated by systematically masking local regions and measuring the resulting change in model output, thereby identifying areas most influential to the classification decision.

Specifically, for each sample and each anatomical plane, a square occlusion window of size 15 × 15 pixels was systematically moved across the selected slice with a stride of five pixels. Within each window, pixel intensities were replaced by the corresponding values from a neutral representative image—defined as a sample whose predicted logit was closest to zero—and the perturbed image was passed through the fixed trained CNN model to obtain a new prediction.

Based on this procedure, for each individual subject *n*, the raw occlusion score at position (*i, j*) was defined as:


$$\begin{array}{l} \delta_{n}\left(i,j\right)=\frac{\sum_{p\in P\left(i,j\right)}f\left(x_{n}\right)-f\left(x_{n}^{\left(p\right)}\right)}{\left|P\left(i,j\right)\right|} \end{array}$$ (2)

where *f*(*x*_*n*_) denotes the logit of the original image and $f\left(x_{n}^{\left(p\right)}\right)$ denotes the logit after occluding patch *p*, *P*(*i, j*) denotes the set of all patches covering pixel (*i, j*), and |*P*(*i, j*)| denotes the number of patches covering pixel (*i, j*).

To ensure that positive scores consistently indicate regions important for correct classification regardless of sex label, a sign-adjusted score was defined as:


$${\tilde{\delta }}_{n}(i,j)=\{\begin{array}{c} \delta_{n}(i,j),if\;subject\;n\;is\;a\;male\;\\ -\delta_{n}(i,j),\;if\;subject\;n\;is\;a\;female \end{array}$$ (3)

For each subject *n*, the top 5% of ${\tilde{\delta }}_{n}\left(i,j\right)$ values were retained to identify the most spatially influential locations. Group-level attribution patterns were then summarized by computing a frequency map:


$$\begin{array}{l} F\left(i,j\right)=\frac{1}{N}\overset{N}{\underset{n=1}{\sum }}I\left({\tilde{\delta }}_{n}\left(i,j\right)\geq \tau_{n}\right) \end{array}$$ (4)

where *N* is the total number of subjects and τ_*n*_ is the subject-specific 95th percentile threshold. The resulting frequency maps were smoothed using a Gaussian kernel (σ = 1.0) for visualization.

After generating occlusion attribution maps for all three anatomical planes, the high-attribution regions within each plane were examined, and a candidate three-dimensional ROI was defined as the spatial location that showed the most consistent high attribution across planes.

To validate the candidate ROI, a lightweight three-dimensional CNN was trained on 24 × 24 × 24 voxel patches extracted from each sample's MRI at the candidate ROI location. A negative control region of identical size was selected from a low-attribution area identified from the same occlusion frequency maps. An equivalent 3D CNN was independently trained on patches extracted from this control region under identical architecture, training settings, and data splits. Classification performance between the two regions was compared using MCC across repeated runs, with the hypothesis that the candidate ROI would yield substantially higher discriminative performance than the negative control.

## 3 Results

### 3.1 Model training and hyperparameter optimization

Based on hyperparameter optimization, the final configuration consisted of weight decay = 0.1, StepLR scheduling with step size = 10, and the inclusion of batch normalization layers. Under this configuration, the sagittal mid-slice CNN model achieved a mean MCC of 0.546 ± 0.010 across ten independent runs, with consistent performance across repetitions. A complete summary of hyperparameter optimization results is provided in Supplementary Table S2.

### 3.2 Simulation study: sensitivity to localized signals

To examine whether CNN can identify spatially localized sex-related information, we conducted a simulation experiment across slices 5–65 in all three anatomical planes. The cubic region ([25:34]^3^) was specifically modified to introduce artificial inflated signals, while all other slices remained unaltered.

In the original dataset, the between-class difference within the cubic region was not statistically significant (*p* = 0.771, energy distance test (Rizzo and Székely, 2016), indicating negligible intrinsic contrast. After simulation, the same region exhibited a significant difference (*p* < 0.05), confirming that the manipulation successfully injected a detectable signal.

**Figure 2:**
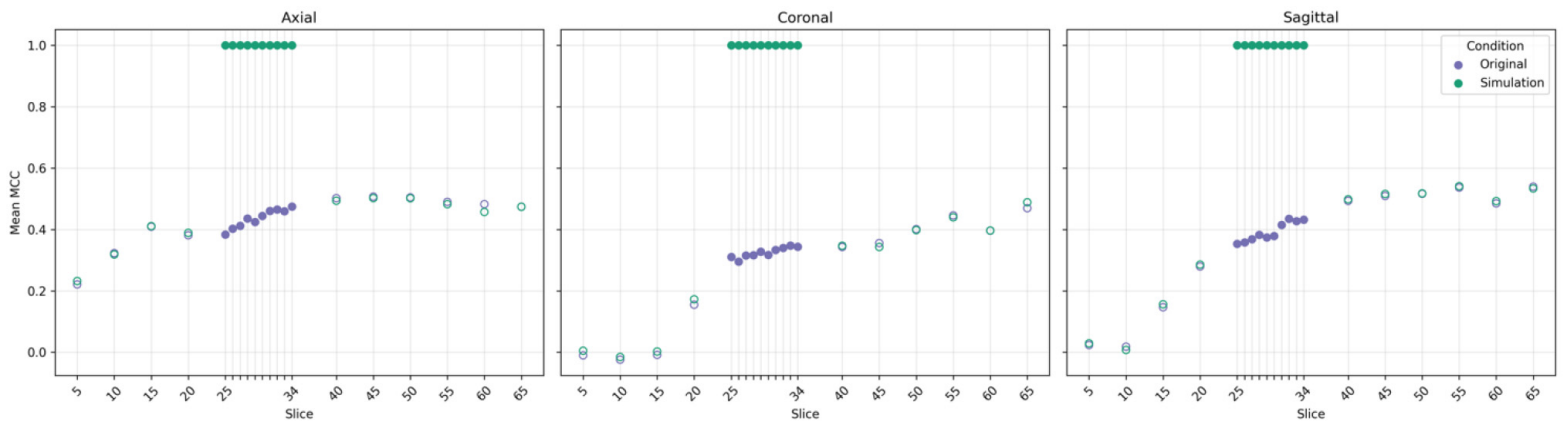
Slice-wise model performance before and after signal simulation. Mean MCC values across slices 5–65 are shown for the axial, coronal, and sagittal planes under the original (purple) and simulation (green) conditions. Hollow markers represent off-cubic slices, whereas solid markers indicate the on-cubic slices (25–34) where the artificial sex-linked signals were injected.

Consistent with this change, Figure 2 shows a clear contrast between unmodified and simulated slices. For unmodified slices (5–24 and 35–65), the mean MCC profiles of the original and simulated datasets overlapped closely across all three anatomical planes, indicating no appreciable change in model performance outside the manipulated region. In contrast, within the simulated cubic region (slices 25–34), a marked performance shift was observed following signal injection. Across all planes, the mean MCC increased sharply to 1.00 with zero variance across runs, whereas no comparable change was observed in adjacent slices. The full slice-wise MCC values summarized in Supplementary Table S3 confirm that this performance increase and variance collapse were strictly confined to the injected region.

### 3.3 Permutation analysis results

To verify that the sex-specific signal learned by the CNN model was not a result of random fluctuations, we performed a permutation experiment in which sex labels were randomly shuffled while keeping all other conditions identical.

**Figure 3:**
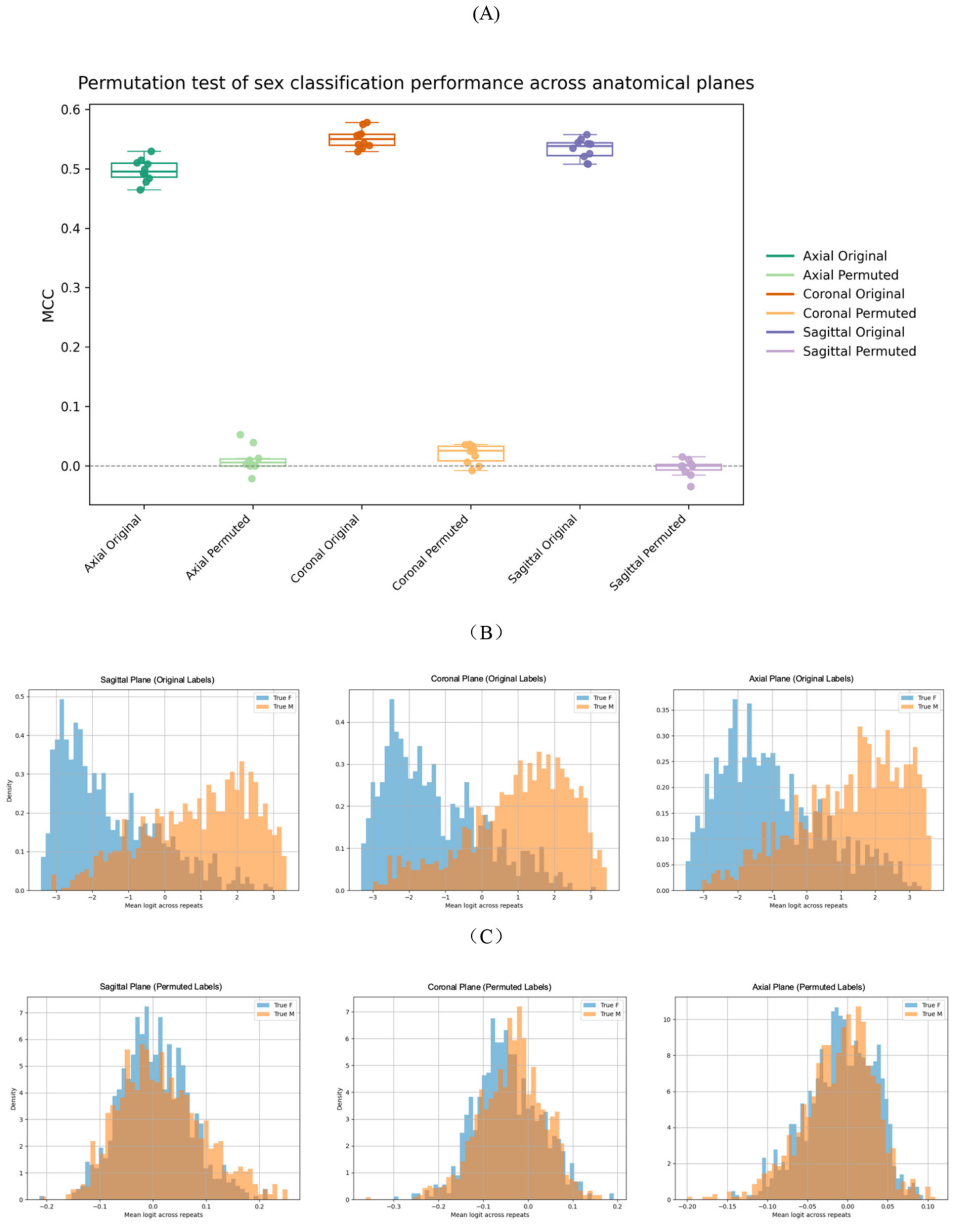
Permutation analysis of CNN performance and logit distributions. **(A)** Comparison of MCC values across anatomical planes for slice-based models trained with true and permuted labels. **(B)** Logit distributions for male and female participants under the original labeling condition, shown separately for sagittal, coronal, and axial planes. **(C)** Corresponding logit distributions under label permutation, illustrating the loss of class separability across all anatomical planes.

Figure 3A shows the comparison of MCC values between models trained with true and permuted labels across anatomical planes. For each plane, as expected, models trained on the original labels consistently achieved substantially higher MCC values than those trained on permuted labels. In contrast, the MCC values obtained under permutation remained close to zero across all planes, indicating a near-complete loss of discriminative ability when label information was randomized.

Figure 3B further illustrates this contrast at the logit level. Under the original-label condition, the distributions of model logits for male and female samples were clearly separated, reflecting effective class discrimination. Under the permuted-label condition, however, the logit distributions for the two classes largely overlapped, indicating that the model failed to separate the classes when trained with randomized labels Figure 3C.

To evaluate whether performance differences between models trained with true and permuted labels were consistently observed across anatomical planes and confidence thresholds, we conducted two-sample *t*-tests for each plane–threshold combination, with threshold definitions specified in Section 2.4. Quantitative results and corresponding visualizations are summarized in Supplementary Table S4 and Supplementary Figure S1. All comparisons showed that models trained with true labels significantly outperformed their permuted-label counterparts (*p* < 0.05).

### 3.4 Confidence-based threshold analysis

To characterize the trade-off between classification performance and data coverage under increasing confidence thresholds, we conducted a dual-threshold analysis that retained only predictions with probabilities below a lower bound or above a corresponding upper bound (e.g., ≤ 0.05 or ≥0.95), thereby excluding intermediate-confidence cases.

**Figure 4:**
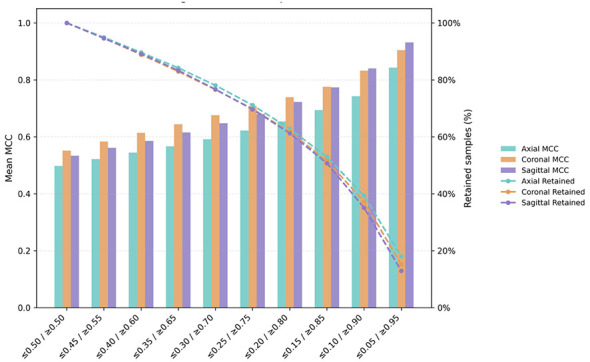
Confidence-based dual-threshold analysis of sex classification performance and data retention across anatomical planes. Mean Matthews correlation coefficient (MCC; left *y*-axis, bars) and the proportion of retained test samples (right *y*-axis, dashed lines) are shown as functions of dual confidence thresholds. For each threshold pair, only predictions with posterior probabilities below the lower bound or above the upper bound were retained. Results are shown separately for axial (cyan), coronal (orange), and sagittal (purple) planes.

As shown by the bar plots in Figure 4, classification performance increased monotonically as the confidence threshold became more stringent across all anatomical planes. Mean MCC values rose steadily from approximately 0.5 under lenient thresholds to above 0.8 under the most stringent settings. Across thresholds, the coronal and sagittal models consistently achieved slightly higher MCC values than the axial model.

Conversely, the line plots in Figure 4 illustrate a progressive reduction in the proportion of retained samples as the confidence thresholds became more stringent. The retention curves for the three anatomical planes largely overlapped, indicating comparable confidence distributions across views. Notably, the decline became markedly steeper beyond thresholds of approximately ≤ 0.20 or ≥0.80, and under the most stringent criteria, only a very small fraction of test samples (10–20% of test samples per plane) was retained.

### 3.5 Spatial localization of sex-linked signals

To locate brain regions showing sex-specific differences from brain MRI, we conducted a stepwise analysis that progressed from identifying the highest-performing slices within each anatomical plane to constructing a candidate three-dimensional reference coordinate.

**Figure 5:**
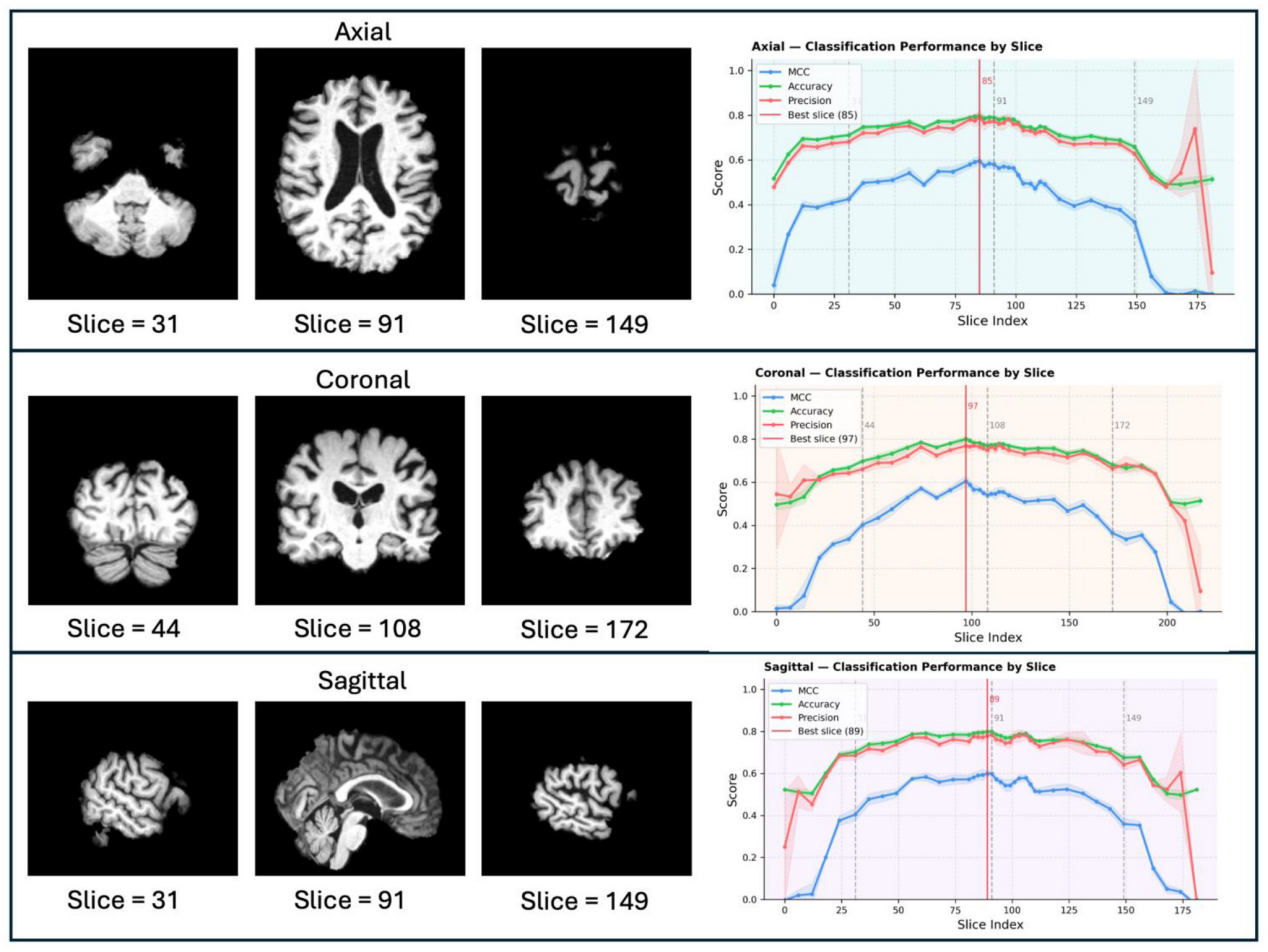
Slice-wise sex classification performance across anatomical planes. For each plane (axial, coronal, and sagittal), representative MRI slices are displayed on the left to provide anatomical context, and the corresponding slice-wise classification metrics are shown on the right. Solid curves denote mean MCC, accuracy, and precision across repeated runs, with shaded bands indicating ±1 standard deviation. Vertical dashed lines mark the slice indices corresponding to the displayed MRI slices; the red vertical line indicates the best-performing slice with the highest mean MCC.

Slice-wise classification performance across anatomical planes is summarized in Figure 5 and Supplementary Tables S5–S7. For each sampled slice, the mean MCC was computed to generate continuous performance profiles along the sagittal, coronal, and axial orientations. Across all three planes, discriminative performance was minimal at peripheral slices and increased toward more central locations.

The slice achieving the highest mean MCC was identified independently in each anatomical plane, corresponding to slice 89 in the sagittal plane (MCC: 0.599 ± 0.012; precision: 0.779 ± 0.014; accuracy: 0.799 ± 0.006), slice 97 in the coronal plane (MCC: 0.605 ± 0.026; precision: 0.769 ± 0.030; accuracy: 0.800 ± 0.014), and slice 85 in the axial plane (MCC: 0.596 ± 0.015; precision: 0.790 ± 0.043; accuracy: 0.797 ± 0.009).

**Figure 6:**
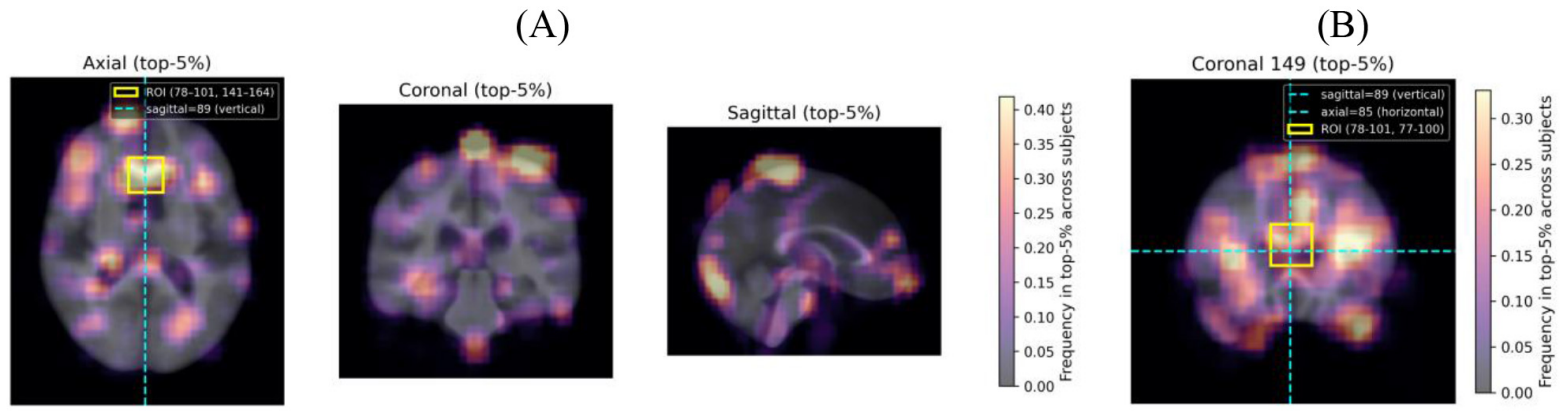
Occlusion-based attribution frequency maps for the three best-performing slices (axial slice 85, coronal slice 97, sagittal slice 89) and an independent coronal validation slice (slice 149). **(A)** In the axial plane, the cyan dashed line indicates the position of the best-performing sagittal slice (slice 89), and the yellow rectangle outlines the projection of the candidate three-dimensional ROI (dim0 = 78–101, dim1 = 141–164, dim2 = 77–100). **(B)** Occlusion-based attribution frequency map for coronal slice 149, which falls within the coronal extent of the candidate ROI (dim1 = 141–164). The cyan dashed lines indicate the positions of sagittal slice 89 (vertical) and axial slice 85 (horizontal), and the yellow rectangle marks the projected ROI boundaries (dim0 = 78–101, dim2 = 77–100).

To further localize spatially discriminative regions, occlusion-based attribution analysis was performed on each of the three best-performing slices (Figure 6A). When identifying candidate regions, peripheral areas of the frequency maps were excluded as they are more likely to reflect skull or background signal rather than genuine brain structure. Under this criterion, the axial plane (slice 85) revealed a spatially concentrated high-attribution region in the upper-central portion of the slice.

To corroborate this localization across independent viewing planes, occlusion-based attribution was additionally examined for the best-performing sagittal slice (slice 89) and coronal slice 149. Notably, sagittal slice 89 passes through the high-attribution region identified in the axial plane, meaning that the slice position itself spatially intersects the candidate region, providing geometric corroboration from an orthogonal viewing angle. Similarly, coronal slice 149 (Figure 6B)—which falls within the coronal extent of the candidate region—yielded elevated attribution concentrated in the area corresponding to the same spatial coordinates, further reinforcing the localization.

Based on this converging evidence across all three planes, a candidate three-dimensional ROI of 24 × 24 × 24 voxels was defined (dim0: 78–101, dim1: 141–164, dim2: 77–100). This region corresponds anatomically to the anterior cingulate cortex and adjacent medial structures including portions of the corpus callosum genu, as visualized across ten representative subjects in Supplementary Figure S5 and in the context of the attribution maps in Figures 6A,B.

To validate that the candidate ROI carries genuine sex-discriminative signal, a 3D CNN was trained on 24 × 24 × 24 voxel patches extracted from the candidate ROI (dim0: 78–101, dim1: 141–164, dim2: 77–100) and compared against a negative control region of identical size located in a low-attribution area (dim0: 79–102, dim1: 54–77, dim2: 35–58; Supplementary Figure S2). The candidate ROI yielded substantially higher classification performance (MCC: 0.457 ± 0.019; accuracy: 0.729 ± 0.009; precision: 0.721 ± 0.016) compared to the negative control region (MCC: 0.304 ± 0.022; accuracy: 0.650 ± 0.011; precision: 0.621 ± 0.014), confirming that the identified region contains spatially localized information relevant to sex classification.

### 3.6 Multi-slice classification

To further assess whether the best-performing slices across planes capture complementary and high-quality discriminative information, we constructed a multi-slice CNN-based classification model taking the three best-performing slices as input for joint classification. The model achieved an MCC of 0.719 ± 0.011, an accuracy of 0.859 ± 0.007, and a precision of 0.856 ± 0.020, demonstrating that the slices identified by the proposed pipeline retain strong discriminative information. The performance gain over individual single-plane models (axial: MCC = 0.596 ± 0.015; coronal: MCC = 0.605 ± 0.026; sagittal: MCC = 0.599 ± 0.012) further suggests that the selected slices across planes contain complementary information.

### 3.7 Correlation between MCC with occlusion-based attribution and GradCAM values

It is of interest to compare slice-based MCC values with well-established region-based discriminative measures such as occlusion-based attribution values and GradCAM values. To do this, we calculated all the occlusion scores for each slice, and GradCAM scores and compared them with MCCs calculated from SliceMap. The results are summarized in Supplementary Figures S6–S8.

These results show that MCC and occlusion-based attribution values are largely correlated across all three anatomical planes (axial: *r* = 0.59, coronal: *r* = 0.64, sagittal: *r* = 0.58, all *p* < 0.001). MCC–GradCAM consistency was strong in the coronal and sagittal planes (*r* = 0.65 and *r* = 0.51, both *p* < 0.001), but not so much in the axial plane (*r* = 0.15, *p* = 0.364). Besides, all three measures show roughly analogous patterns in the curve profiles across all three planes: MCC varies smoothly across slices, whereas occlusion and GradCAM scores exhibit greater slice-to-slice fluctuation. This variation likely reflects a fundamental difference in what each metric captures. MCC is computed on the full image and therefore integrates global discriminative information across the entire slice. In contrast, both occlusion and GradCAM scores calculated in this study are summarized as the per-subject maximum activation—retaining only the single most salient local region—which renders them more sensitive to spatially concentrated signals and thus inherently more variable across slices.

Together, these results suggest that MCC and the attribution scores are complementary rather than redundant: MCC characterizes overall slice-level predictive utility, while occlusion and GradCAM scores highlight the local regions that most strongly drive the model's predictions.

## 4 Discussion

In this study, we proposed a two-dimensional (2D) slice-based pipeline for detecting discriminative candidate regions in binary classification tasks using brain MRI data. The pipeline is computationally efficient and designed with broad applicability in mind, offering a practical alternative to fully three-dimensional modeling approaches when large-scale or iterative analyses are required. The proposed framework is built upon plane-specific 2D convolutional neural networks, and sex classification was adopted as a controlled testbed to demonstrate and validate the pipeline.

### 4.1 Validation of detected region

The candidate ROI identified by the proposed pipeline occupies voxel coordinates in slices 78–101 in sagittal plane, slices 141–164 in coronal plane, and slices 77–100 in axial plane of the preprocessed image space, corresponding anatomically to the anterior cingulate cortex (ACC) and adjacent medial structures including portions of the corpus callosum genu.

The identification of this region followed a two-step process, each with a distinct source of evidence. In the first step, the best-performing slice from each anatomical plane was selected based on performance measure MCC, providing a data-driven coordinate reference in 3D space. The reliability of this selection is supported by the simulation and permutation experiments. The simulation experiment demonstrated that the 2D CNN is sensitive to spatially localized discriminative signals: when an artificial signal was injected into a known cubic region, slice-wise MCC was selectively elevated at slices intersecting that region, confirming that high MCC reflects genuine spatial signals. The permutation experiment further confirmed that the observed classification performance reflects learned biological structure rather than spurious correlations, as models trained on permuted labels showed significantly lower MCC than those trained on true labels. In the second step, occlusion-based attribution analysis was applied to each best-performing slice. The occlusion approach is methodologically transparent: by systematically masking local regions and measuring the resulting change in model output, the contribution of each spatial location is directly quantified without relying on gradient approximations or architectural assumptions, making the resulting attribution maps readily interpretable.

Critically, to further quantify the discriminative content of the identified region, a 3D CNN was trained on the selected 24 × 24 × 24 high-attribution candidate ROI and compared against a size-matched negative control region located in a low-attribution area. The candidate ROI yielded substantially higher classification performance (MCC: 0.457 ± 0.019; accuracy: 0.729 ± 0.009; precision: 0.721 ± 0.016) compared to the negative control (MCC: 0.304 ± 0.022; accuracy: 0.650 ± 0.011; precision: 0.621 ± 0.014), providing quantitative evidence that the identified region contains spatially localized sex-discriminative information.

The anatomical location of the candidate ROI is also consistent with prior neuroimaging literature reporting sex differences in the ACC and corpus callosum genu. Structural MRI studies have consistently reported proportionally greater ACC volume in females than in males across multiple independent samples. Multiple VBM studies have found proportionally greater gray matter volumes in the anterior cingulate region in women compared to men (Brun et al., 2009). A large-scale comparative neuroimaging study further confirmed that the anterior cingulate cortex is significantly larger in females bilaterally in both humans and mice (Guma et al., 2024), suggesting this sex difference may reflect a conserved biological pattern. Morphometric analyses have additionally identified sex differences in the gross morphology of the ACC, with males showing greater asymmetric fissurization of the left ACC and females showing greater hemispheric symmetry (Yücel et al., 2001). Furthermore, a prominent right anterior cingulate gyrus has been reported to be more frequent in women than in men (Pujol et al., 2002), with this structural difference linked to sex differences in personality traits related to harm avoidance. Regarding the corpus callosum genu, prior MRI studies have reported that the midsagittal corpus callosum area is significantly larger in females after controlling for brain size, with the genu showing among the most pronounced sex-related differences (Shiino et al., 2017; Ardekani et al., 2013). Taken together, these findings support the biological plausibility of a midline region encompassing the anterior cingulate cortex (ACC) and adjacent medial structures, including portions of the corpus callosum genu, as a locus of sex-linked structural variation detectable in our study.

### 4.2 Brain tissue coverage and slice discriminability

The slice-wise MCC curves reveal a consistent pattern across all three anatomical planes: slices sampled from central brain regions, which contain greater tissue coverage, tend to achieve higher classification performance than peripheral slices. This observation suggests that brain tissue coverage may partially contribute to slice-level discriminability, likely because more tissue provides a richer representation of structural features relevant to the classification task.

However, tissue coverage alone does not fully account for the observed performance differences. To disentangle the contribution of tissue quantity from that of anatomical content, we conducted a controlled comparison using the best-performing axial and coronal slices (see Supplementary Section SA). Each optimal slice was divided into left and right hemispheric halves, reducing tissue coverage by approximately half. Despite this substantial reduction in tissue area, classification performance did not drop proportionally—both hemispheric halves retained a substantial portion of the discriminative ability of the full slice. Furthermore, when compared against peripheral slices with comparable or even greater nonzero pixel counts, the hemispheric halves consistently achieved higher MCC (Supplementary Figure S3, Supplementary Table S8). This two-part dissociation—performance degrades less than tissue area, and outperforms size-matched peripheral slices—indicates that the performance advantage of the identified optimal slices reflects anatomically specific discriminative content rather than tissue quantity alone.

These findings suggest that the optimal slices selected by the proposed pipeline capture anatomically specific discriminative content that goes beyond tissue abundance. The occlusion-based attribution maps revealed localized high-attribution regions within these slices, which guided the identification of the candidate ROI in the anterior cingulate and surrounding medial frontal regions. The subsequent 3D CNN validation, in which the candidate ROI substantially outperformed the size-matched control region (Section 2.5), provides supporting evidence that the performance advantage of the identified slices is driven by spatially concentrated sex-linked structural variation rather than tissue quantity alone.

### 4.3 Applicability of the pipeline to other tasks

The proposed pipeline is designed to be task-agnostic and can in principle be applied to any binary classification problem in neuroimaging where the goal is to identify candidate discriminative regions. The core components—slice-wise CNN training, performance-guided slice selection, and occlusion-based attribution—do not rely on any assumptions specific to sex classification, and can be straightforwardly adapted to other binary contrasts of interest.

To probe this generalizability, we applied the pipeline to the task of distinguishing Alzheimer's disease (AD) from cognitively normal (CN) subjects, as described in Supplementary Section SB. The resulting classification performance was modest across all three anatomical planes, with notably high variance across repeated runs in certain slices, indicating that the models failed to learn stable and reliable discriminative features. We attribute this to several compounding factors specific to the AD/CN dataset. First, because the Research Group label in ADNI reflects disease status only at the baseline visit, analyses were restricted to baseline visits, substantially reducing the available sample size compared to the sex classification task. Second, controlling for confounders such as sex and age through subject matching further constrained the usable data to 371 matched pairs. Third, with such limited sample sizes, the train/validation/test split cannot guarantee comparable class distributions across subsets, which may destabilize model learning and reduce the reliability of downstream performance estimates.

These limitations do not reflect a fundamental constraint of the pipeline itself, but rather highlight the practical requirement for sufficient and high-quality data. We expect that with a larger and more balanced AD/CN dataset, the pipeline would yield more stable slice-wise performance and support meaningful region localization.

### 4.4 Limitations and future work

Several limitations of the present study motivate future work.

First, although confidence-based thresholding substantially improved predictive reliability, localization analyses were performed without explicitly conditioning on prediction confidence. Future studies could incorporate confidence-aware localization strategies to assess whether restricting analyses to high-confidence samples yields more consistent or focal spatial patterns.

Second, the modest performance observed in the AD/CN application highlights the critical role of sample size in the proposed pipeline. With only 371 matched pairs available after restricting to baseline visits and controlling for confounders, the models showed unstable learning and high cross-run variance. Future work could address this by aggregating data across multiple neuroimaging databases to construct larger cohorts. Alternatively, leveraging pretrained model weights from existing studies on related classification tasks and fine-tuning on the target dataset may offer a practical strategy for improving performance in data-limited disease classification settings.

Third, the multi-view fusion model combining the best-performing slices from all three anatomical planes achieved substantially higher classification performance than any single-plane model, suggesting that complementary discriminative information is distributed across anatomical views. In the current pipeline, each plane contributed a single slice selected independently based on single-plane MCC, without optimizing for joint discriminative performance across planes. A natural extension would be to systematically enumerate combinations of candidate slices across planes and select the combination that maximizes fusion model performance, then apply occlusion-based attribution to the resulting model—potentially yielding a more reliable basis for spatial localization than attribution applied to independently selected single-plane models.

Fourth, the moderate to strong correlations between MCC and attribution scores observed across almost all planes (Supplementary Figures S6–S8) suggest that MCC-based slice selection serves as a reasonable surrogate for attribution-based localization, supporting its use as a principled and computationally efficient criterion. However, the slice with the highest MCC does not necessarily coincide with the slice exhibiting the highest occlusion-based or GradCAM attribution score, indicating that the two criteria capture partially distinct aspects of discriminative information. Future work could explore whether directly using attribution-based scores as the slice-selection criterion yields more spatially focused and biologically interpretable candidate regions.

Finally, while the proposed 2D pipeline is computationally efficient, a systematic comparison with fully three-dimensional approaches remains an important open question. In the present study, Dropout (rate = 0.5) was employed as the primary regularization strategy to mitigate overfitting given the available sample size. However, extending the pipeline to a 3D volumetric architecture with the same dataset would introduce substantially greater parameter complexity, and whether such a model could be stably trained without severe overfitting remains an open question. Prior work has shown that 3D CNN performance continues to improve with larger cohort sizes (Ebel et al., 2023), suggesting that the benefits of volumetric modeling may only be fully realized with substantially larger datasets. Future studies could address this by aggregating data across multiple neuroimaging initiatives to increase both sample size and demographic diversity, which would not only enable more stable 3D model training but also potentially improve the spatial precision of signal localization.

## Data Availability

Publicly available datasets were analyzed in this study. This data can be found at: Alzheimer's Disease Neuroimaging Initiative (ADNI, https://adni.loni.usc.edu).
