# Supplementary material for: SliceMap: a binary classification-driven 2D pipeline for detecting discriminative candidate regions in brain MRI
Source: Front Neuroimaging. 2026 May 15;5:1819355. doi: 10.3389/fnimg.2026.1819355 (PMC13218861; doi:10.3389/fnimg.2026.1819355)
Supplement: Supplementary file 1 [file Table_1.docx]

Supplementary Material

For

SliceMap: A Binary Classification-Driven 2D Pipeline for Detecting Discriminative Candidate Regions in Brain MRI

Xiaoye Jiang^1^, Zhijin Wu^2^, Zhaohui Qin^1*^

^1^Department of Biostatistics and Bioinformatics, Emory University, Atlanta, GA, United States

^2^Department of Biostatistics, Brown University, Providence, RI 02912, United States

## Relationship Between Brain Tissue Coverage and Classification Performance

This section examines the relationship between brain tissue coverage and slice-wise classification performance, with the aim of investigating whether the strong performance observed at the identified optimal slices (axial slice 85, coronal slice 97, and sagittal slice 89) is primarily attributable to their larger tissue coverage rather than anatomically specific discriminative features.

Brain tissue coverage per slice was quantified by counting the number of nonzero pixels. To examine whether tissue size alone explains the observed performance pattern, the best-performing axial and coronal slices were each divided into left and right hemispheres, and independent CNN models were trained on each half. Despite containing approximately half the tissue of the full slice, both hemispheres maintained relatively competitive performance (Table S8).

To control for tissue size more directly, we identified peripheral slices with comparable or greater nonzero pixel counts to the hemispheric inputs. These slices achieved lower MCC values despite their similar or even larger tissue coverage (Figure S3, Table S8). Together, these results suggest that tissue quantity alone is insufficient to explain classification performance, and that anatomical location and content likely play an important role in determining discriminative signal.

1. **Application of the Proposed Pipeline to Alzheimer's Disease Classification**

To potential applicability of the proposed pipeline, we applied it to the task of distinguishing Alzheimer's disease (AD) from cognitively normal (CN) subjects using the same dataset.

Given that the Research Group label in ADNI reflects disease status at the baseline visit only, analyses were restricted to baseline visits. To control for potential confounders, AD and CN subjects were matched by sex and age, yielding 371 matched AD–CN pairs (742 subjects total). Pair IDs were used to ensure subject-level train/validation/test splitting.

Applying the slice-wise CNN pipeline to the matched dataset identified the best-performing slices as axial slice 49 (MCC = 0.285 ± 0.074), coronal slice 134 (MCC = 0.380 ± 0.049), and sagittal slice 91 (MCC = 0.284 ± 0.062), as shown in Figure S4. Given the relatively modest classification performance and high variability across repeated runs, occlusion-based region localization was not performed, as the validity of attribution analysis is contingent on sufficient model discriminability and stability.

## Supplementary Figures


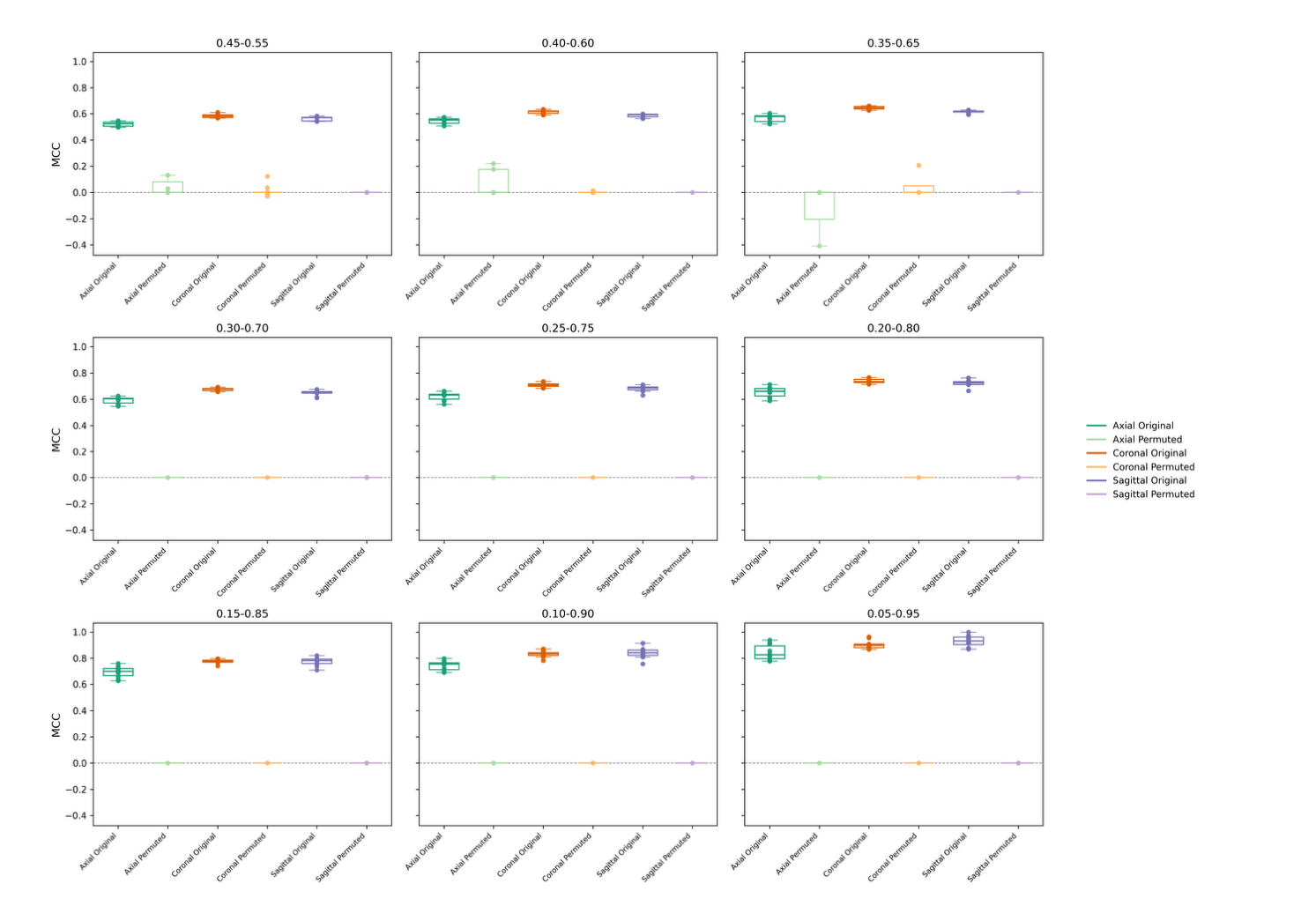


**Figure S1. Permutation test of sex classification performance across thresholds.** Boxplots show MCC distributions for models trained with original and permuted labels across sagittal, coronal, and axial planes under nine alternative threshold settings (excluding the representative threshold of 0.5 shown in the main text). For all thresholds, models trained with true labels consistently outperform their permuted-label counterparts.


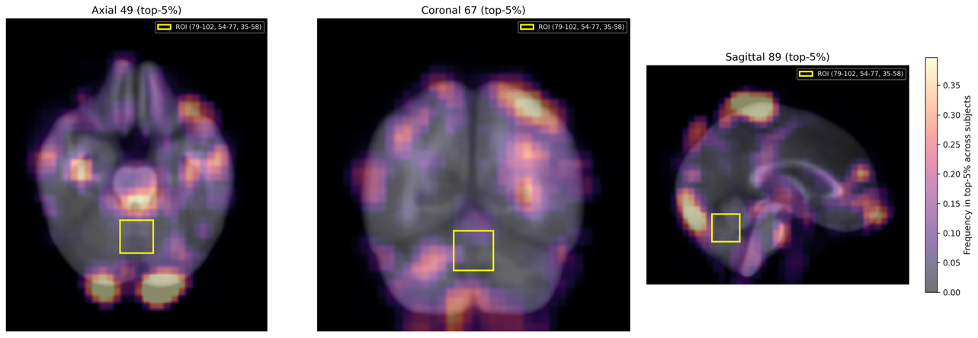
**Figure S2. Occlusion-based attribution frequency maps for the negative control region across three anatomical planes (top-5%).** The yellow rectangle outlines the projection of the negative control region (dim0 = 79–102, dim1 = 54–77, dim2 = 35–58) onto each plane. Slices shown are axial slice 49, coronal slice 67, and sagittal slice 89, corresponding to slices passing the control region.


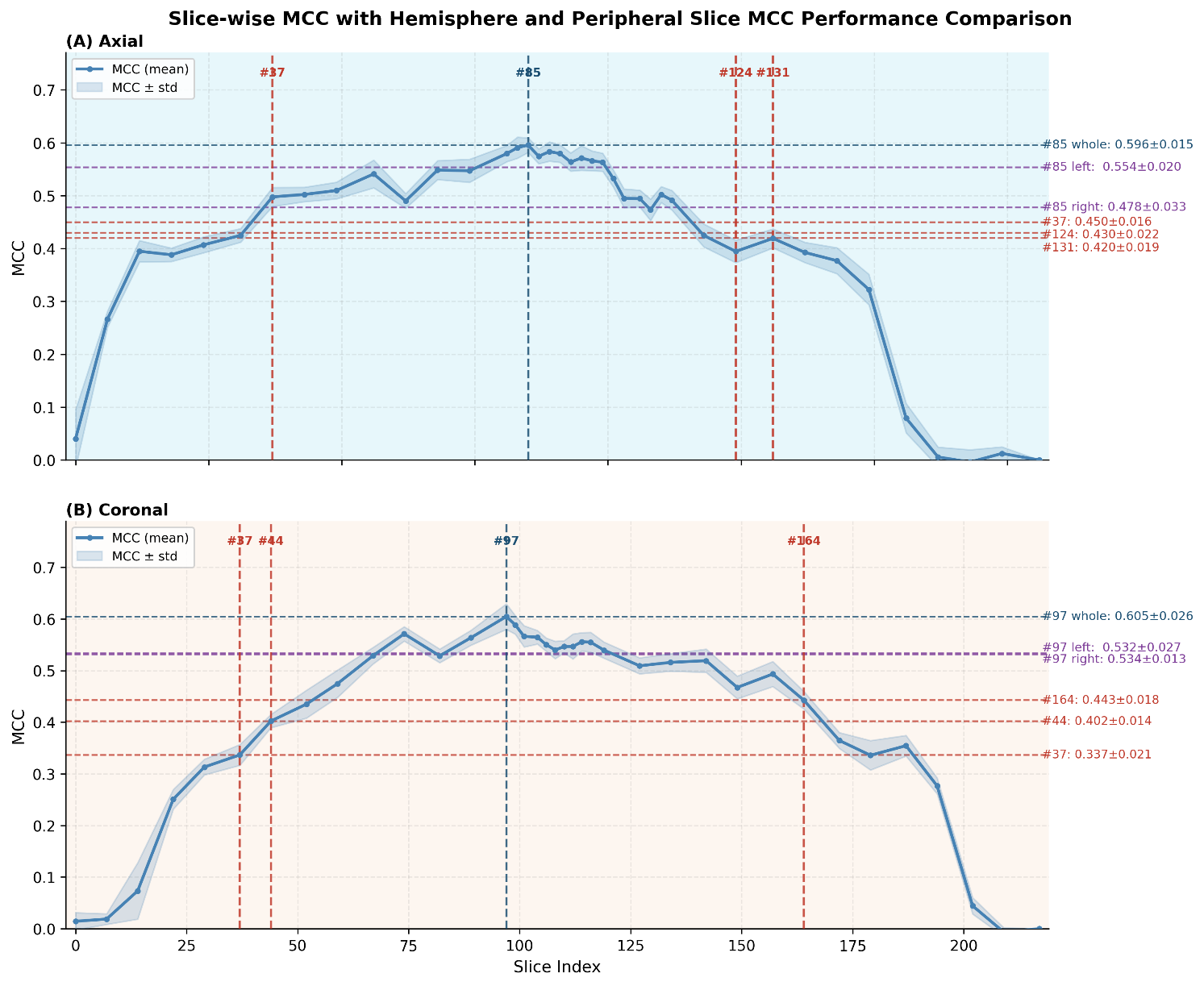
**Figure S3. Slice-wise MCC with hemisphere and peripheral slice comparisons for the axial (A) and coronal (B) planes.** The blue curve shows mean MCC ± standard deviation across 10 repeated runs. Vertical dashed lines indicate the position of specific slices along the x-axis: the navy line marks the best-performing slice, and red lines mark peripheral slices selected for comparison. Horizontal dashed lines indicate the corresponding MCC values: navy for the whole best slice, purple for its left and right hemispheric halves, and red for the peripheral comparison slices.


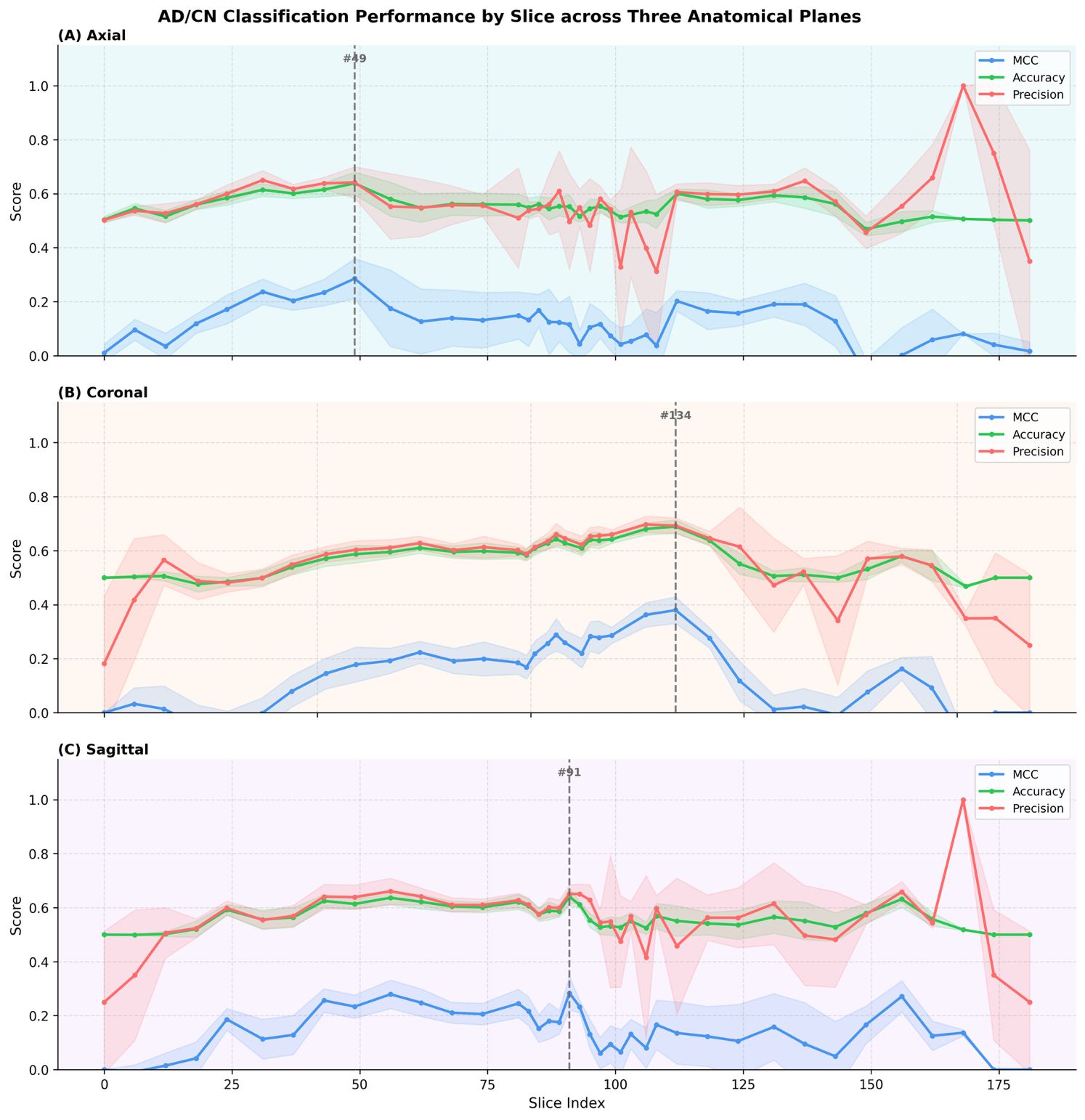
**Figure S4. AD/CN classification performance by slice across three anatomical planes.** Mean MCC, accuracy, and precision across 10 repeated runs are shown for each slice, with shaded regions indicating ± one standard deviation.


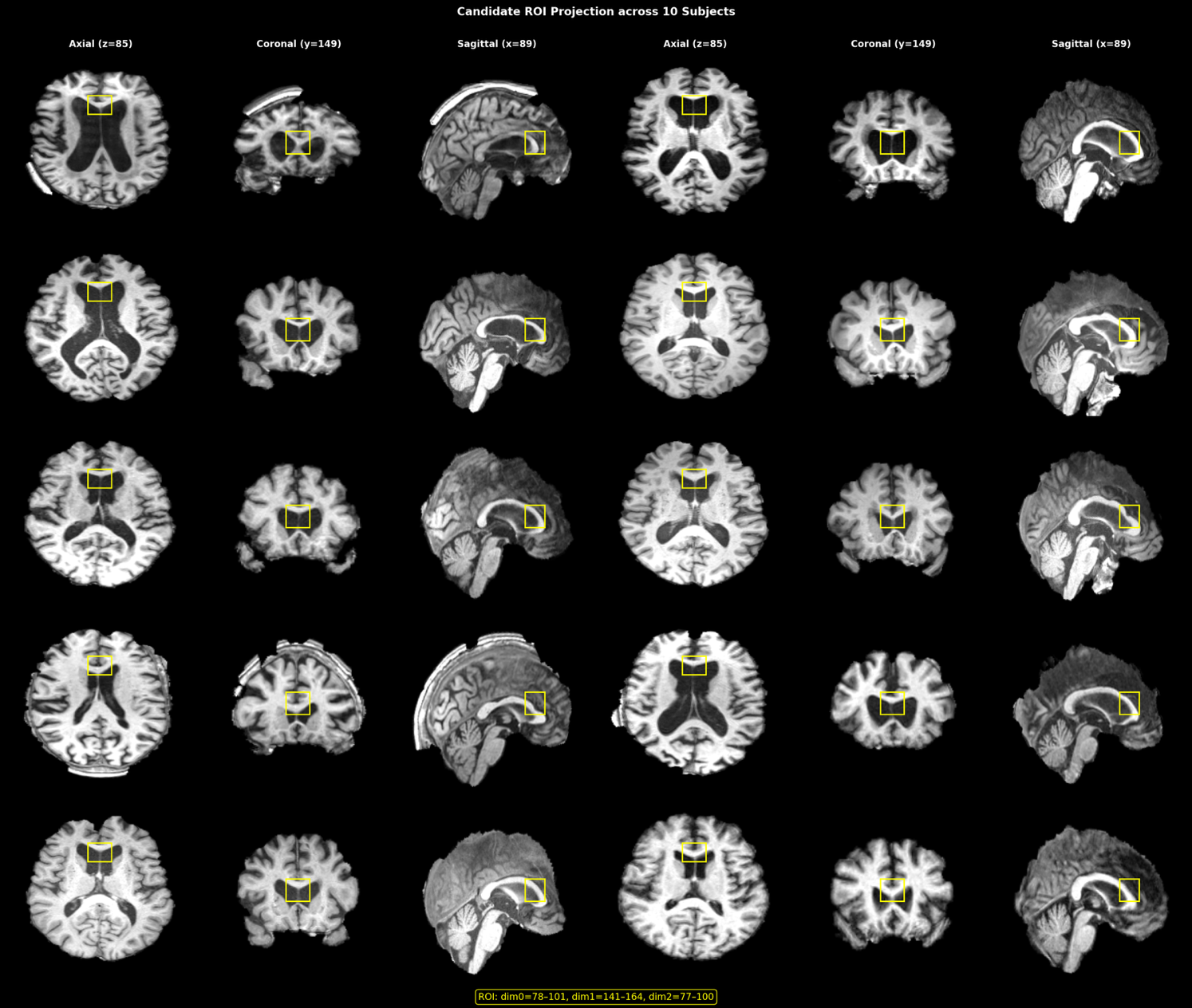
**Figure S5. Candidate ROI projected onto three anatomical planes across ten representative subjects (two subjects per row).** For each subject, axial (z=85), coronal (y=149), and sagittal (x=89) slices are shown. Yellow boxes indicate the candidate ROI (dim0: 78–101, dim1: 141–164, dim2: 77–100), corresponding to the anterior cingulate cortex and adjacent medial structures including portions of the corpus callosum genu.


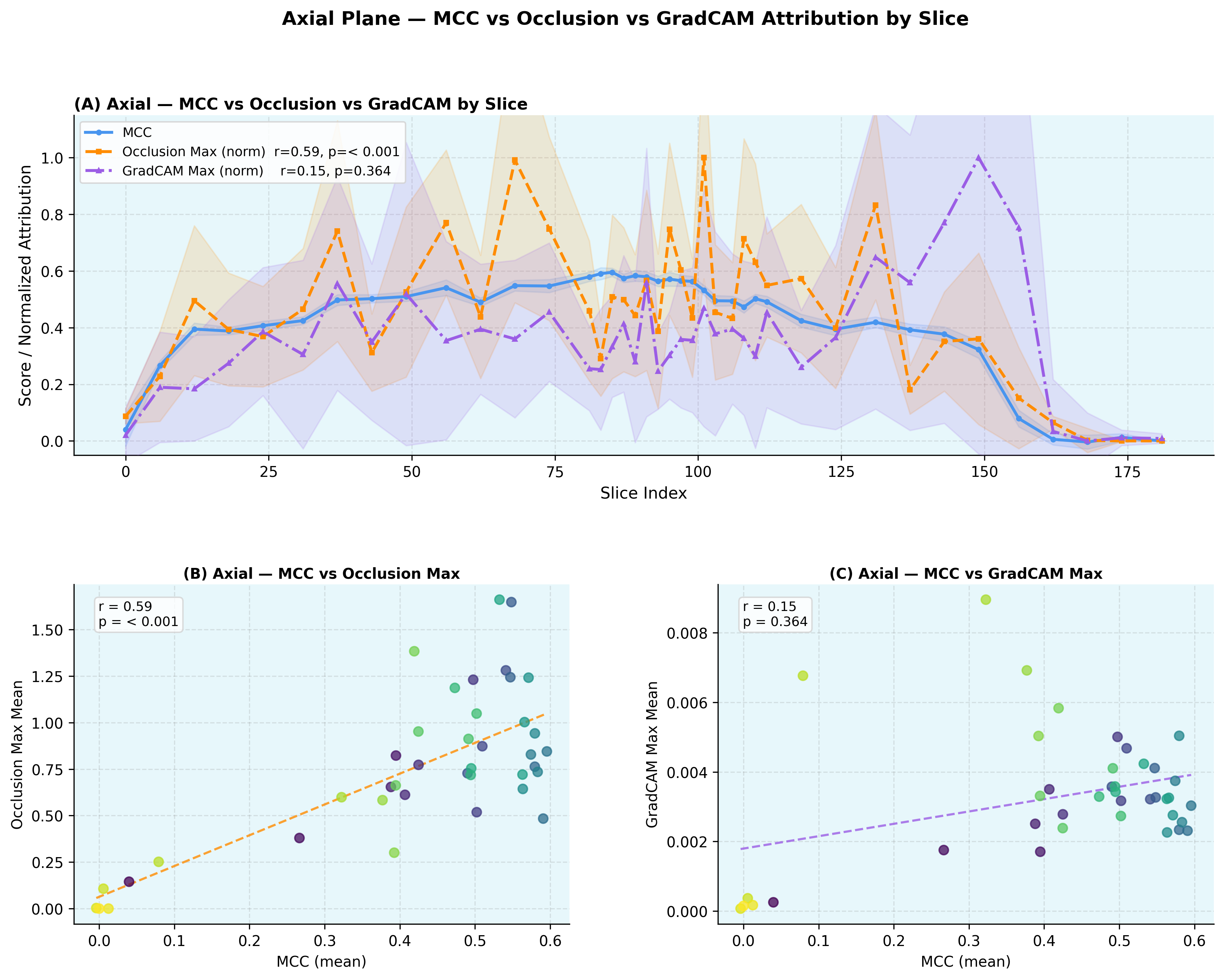
**Figure S6. MCC, occlusion, and GradCAM attribution by slice — axial plane.** (A) Min-max normalized MCC (mean ± SD), occlusion max, and GradCAM max plotted against slice index. Shaded bands indicate ±1 SD. Spearman correlation coefficients (r) and p-values between MCC and each attribution method are shown in the legend. (B) Scatter plot of per-slice mean MCC versus occlusion max mean. Each point represents one slice; the dashed line shows the linear trend. Spearman correlation r and p-value are annotated. (C) Scatter plot of per-slice mean MCC versus GradCAM max mean. Point color encodes slice order (viridis). Spearman correlation r and p-values are annotated.


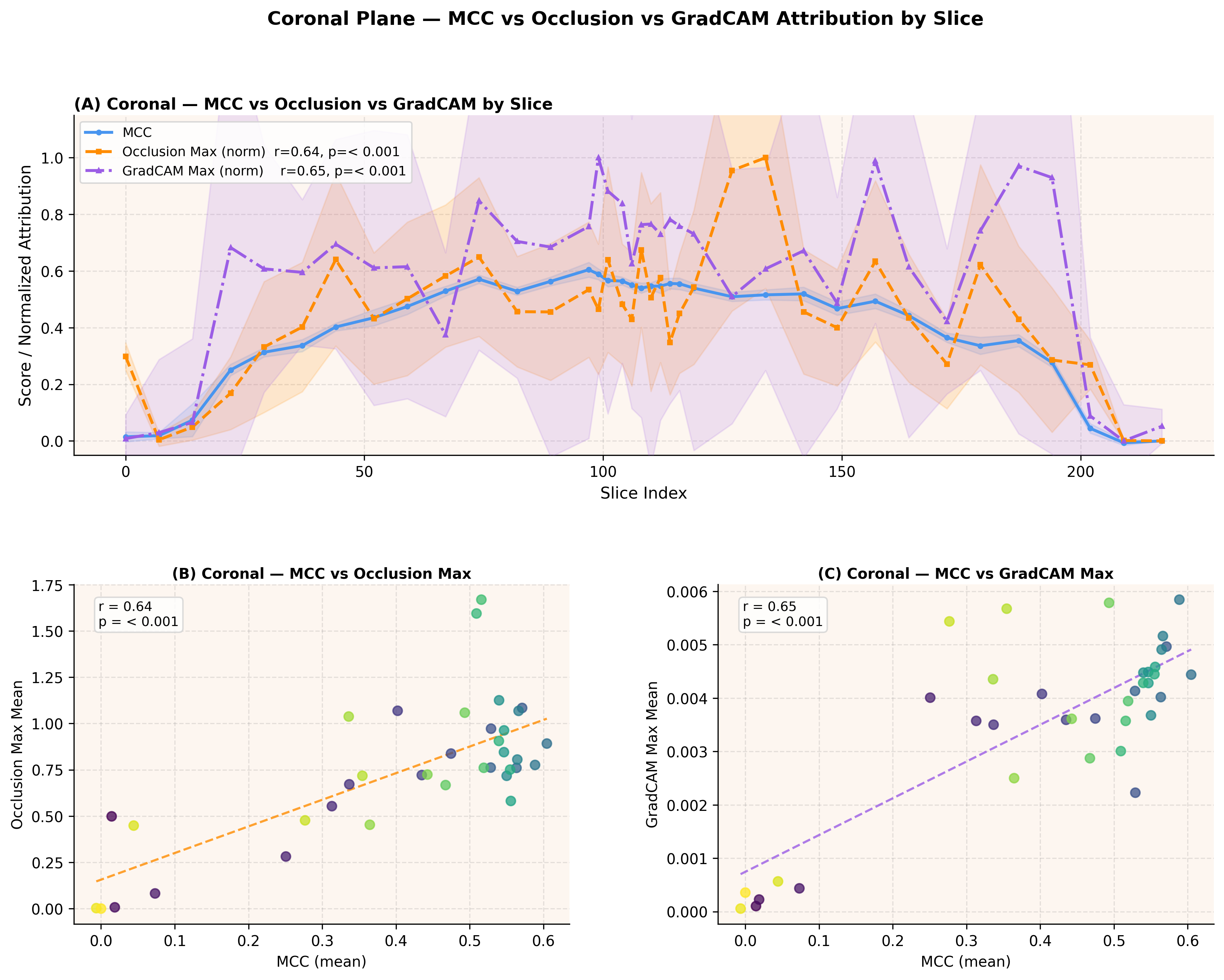
**Figure S7. MCC, occlusion, and GradCAM attribution by slice — coronal plane.** (A) Min-max normalized MCC (mean ± SD), occlusion max, and GradCAM max plotted against slice index. Shaded bands indicate ±1 SD. Spearman correlation coefficients (r) and p-values between MCC and each attribution method are shown in the legend. (B) Scatter plot of per-slice mean MCC versus occlusion max mean. Each point represents one slice; the dashed line shows the linear trend. Spearman correlation r and p-values are annotated. (C) Scatter plot of per-slice mean MCC versus GradCAM max mean. Point color encodes slice order (viridis). Spearman correlation r and p-values are annotated.


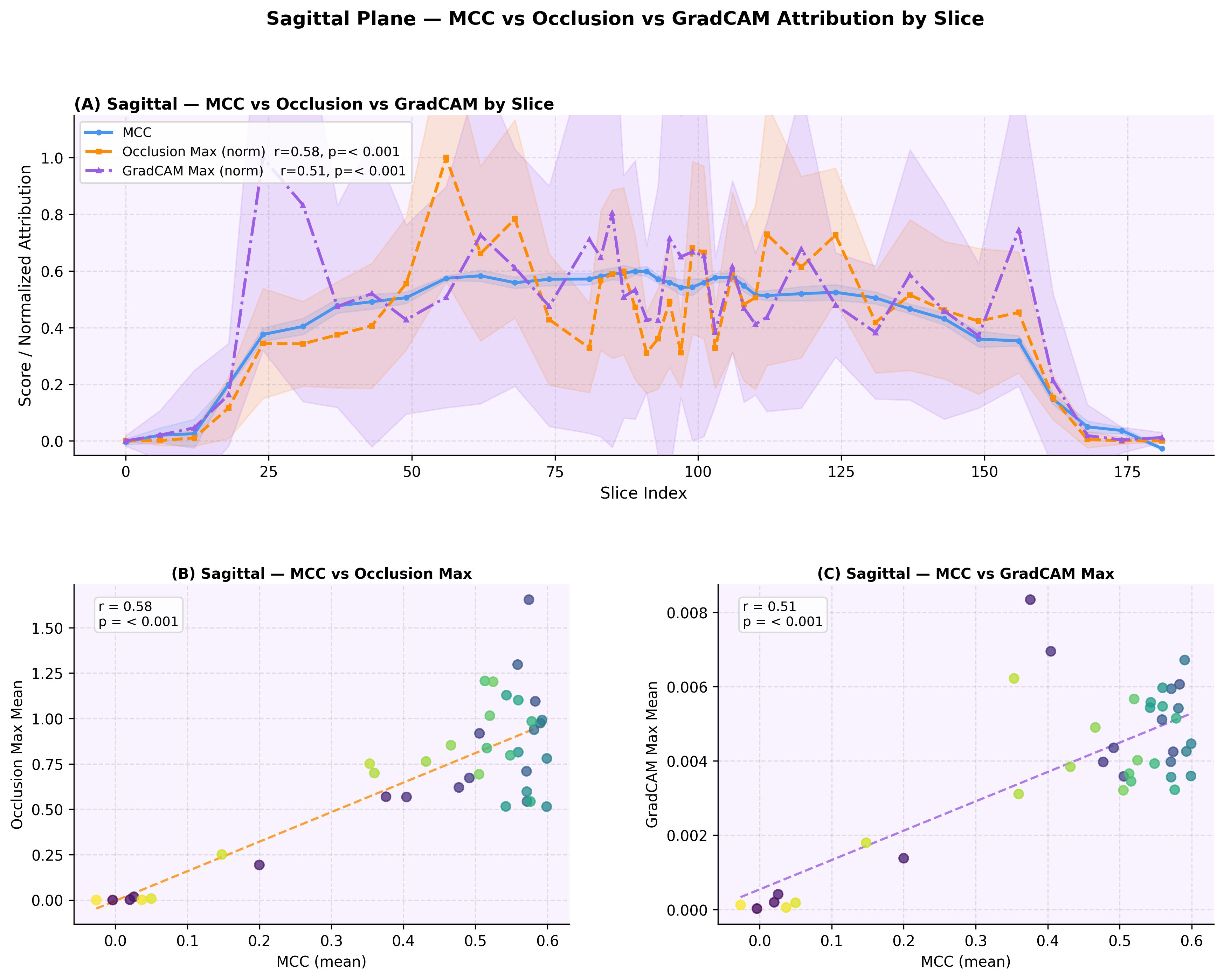
**Figure S8. MCC, occlusion, and GradCAM attribution by slice — sagittal plane.** (A) Min-max normalized MCC (mean ± SD), occlusion max, and GradCAM max plotted against slice index. Shaded bands indicate ±1 SD. Spearman correlation coefficients (r) and p-values between MCC and each attribution method are shown in the legend. (B) Scatter plot of per-slice mean MCC versus occlusion max mean. Each point represents one slice; the dashed line shows the linear trend. Spearman correlation r and p-values are annotated. (C) Scatter plot of per-slice mean MCC versus GradCAM max mean. Point color encodes slice order (viridis). Spearman correlation r and p-values are annotated.

## Supplementary Tables

**Table S1. Demographic summary by sex.** Age is reported as mean$\pm$SD; diagnostic categories are reported as n (column %).

| **Variable** | **Overall (n=9938)** | **Male (n=5379)** | **Female (n=4559)** |
| --- | --- | --- | --- |
| **Age (years), mean**$\boldsymbol{\pm}$**SD** | | | |
| Age | 75.22$\pm7.43$ | 76.08$\pm$7.11 | 74.20$\pm7.67$ |
| **Diagnostic category, n (column %)** | | | |
| CN | 3176 (31.96%) | 1506 (28.00%) | 1679 (36.83%) |
| SMC | 267 (2.69%) | 117 (2.18%) | 150 (3.29%) |
| EMCI | 1416 (14.25%) | 785 (14.59%) | 631 (13.84%) |
| LMCI | 711 (7.15%) | 345 (6.41%) | 366 (8.03%) |
| MCI | 3130 (31.50%) | 1976 (36.74%) | 1154 (25.31%) |
| AD | 1222 (12.30%) | 646 (12.01%) | 576 (12.63%) |
| Patient | 7 (0.07%) | 4 (0.07%) | 3 (0.07%) |

**Table S2. Hyperparameter setting and performance summary.**

| **Weight decay** | **StepLR** | **Step size** | **BatchNorm** | **Mean MCC**$\boldsymbol{\pm}$**SD** |
| --- | --- | --- | --- | --- |
| 0.1 | TRUE | 15 | TRUE | 0.543$\pm0.017$ |
| 0.01 | TRUE | 15 | TRUE | 0.539$\pm0.020$ |
| 0.001 | TRUE | 15 | TRUE | 0.540$\pm0.018$ |
| **0.1** | **TRUE** | **10** | **TRUE** | **0.546**$\boldsymbol{\pm0.010}$ |
| 0.1 | TRUE | 5 | TRUE | 0.543$\pm0.016$ |
| 0.1 | FALSE | / | TRUE | 0.533$\pm0.025$ |
| 0.1 | TRUE | 10 | FALSE | 0.525$\pm0.022$ |

**Table S3. Slice-wise model performance (mean**$\boldsymbol{\pm}$**SD) across three anatomical planes under Original and Simulation conditions.** Slices 25--34 correspond to the cubic region where artificial sex-linked signals were injected.

| **Slice** | **Sagittal (Original)** | **Sagittal (Simulated)** | **Coronal (Original)** | **Coronal (Simulated)** | **Axial (Original)** | **Axial (Simulated)** |
| --- | --- | --- | --- | --- | --- | --- |
| 5 | 0.023$\pm0.010$ | 0.029$\pm0.008$ | -0.010$\pm0.013$ | 0.005$\pm0.017$ | 0.221$\pm0.$017 | 0.232$\pm0.014$ |
| 10 | 0.018$\pm$0.012 | 0.007$\pm0.009$ | -0.024$\pm0.008$ | -0.015$\pm0.017$ | 0.323$\pm0.012$ | $0.318\pm0.018$ |
| 15 | 0.147$\pm0.017$ | 0.156$\pm0.009$ | -0.009$\pm0.010$ | 0.002$\pm0.030$ | 0.409$\pm0.014$ | 0.411$\pm0.012$ |
| 20 | 0.279$\pm0.020$ | 0.285$\pm0.008$ | 0.155$\pm0.024$ | 0.172$\pm0.024$ | 0.381$\pm0.021$ | 0.389$\pm0.022$ |
| **25** | **0.353**$\boldsymbol{\pm0.021}$ | **1.000**$\boldsymbol{\pm0.000}$ | **0.310**$\boldsymbol{\pm0.022}$ | **1.000**$\boldsymbol{\pm0.000}$ | **0.383**$\boldsymbol{\pm0.008}$ | **1.000**$\boldsymbol{\pm0.000}$ |
| **26** | **0.358**$\boldsymbol{\pm0.018}$ | **1.000**$\boldsymbol{\pm0.000}$ | **0.296**$\boldsymbol{\pm0.023}$ | **1.000**$\boldsymbol{\pm0.000}$ | **0.402**$\boldsymbol{\pm0.013}$ | **1.000**$\boldsymbol{\pm0.000}$ |
| **27** | **0.368**$\boldsymbol{\pm0.027}$ | **1.000**$\boldsymbol{\pm0.000}$ | **0.315**$\boldsymbol{\pm0.017}$ | **1.000**$\boldsymbol{\pm0.000}$ | **0.412**$\boldsymbol{\pm0.009}$ | **1.000**$\boldsymbol{\pm0.000}$ |
| **28** | **0.383**$\boldsymbol{\pm0.011}$ | **1.000**$\boldsymbol{\pm0.000}$ | **0.316**$\boldsymbol{\pm0.027}$ | **1.000**$\boldsymbol{\pm0.000}$ | **0.435**$\boldsymbol{\pm0.013}$ | **1.000**$\boldsymbol{\pm0.000}$ |
| **29** | **0.374**$\boldsymbol{\pm0.026}$ | **1.000**$\boldsymbol{\pm0.000}$ | **0.328**$\boldsymbol{\pm0.020}$ | **1.000**$\boldsymbol{\pm0.000}$ | **0.424**$\boldsymbol{\pm0.017}$ | **1.000**$\boldsymbol{\pm0.000}$ |
| **30** | **0.378**$\boldsymbol{\pm0.022}$ | **1.000**$\boldsymbol{\pm0.000}$ | **0.318**$\boldsymbol{\pm0.013}$ | **1.000**$\boldsymbol{\pm0.000}$ | **0.444**$\boldsymbol{\pm0.016}$ | **1.000**$\boldsymbol{\pm0.000}$ |
| **31** | **0.415**$\boldsymbol{\pm0.020}$ | **1.000**$\boldsymbol{\pm0.000}$ | **0.333**$\boldsymbol{\pm0.012}$ | **1.000**$\boldsymbol{\pm0.000}$ | **0.416**$\boldsymbol{\pm0.016}$ | **1.000**$\boldsymbol{\pm0.000}$ |
| **32** | **0.435**$\boldsymbol{\pm0.016}$ | **1.000**$\boldsymbol{\pm0.000}$ | **0.340**$\boldsymbol{\pm0.016}$ | **1.000**$\boldsymbol{\pm0.000}$ | **0.465**$\boldsymbol{\pm0.017}$ | **1.000**$\boldsymbol{\pm0.000}$ |
| **33** | **0.427**$\boldsymbol{\pm0.027}$ | **1.000**$\boldsymbol{\pm0.000}$ | **0.348**$\boldsymbol{\pm0.010}$ | **1.000**$\boldsymbol{\pm0.000}$ | **0.459**$\boldsymbol{\pm0.031}$ | **1.000**$\boldsymbol{\pm0.000}$ |
| **34** | **0.432**$\boldsymbol{\pm0.004}$ | **1.000**$\boldsymbol{\pm0.000}$ | **0.344**$\boldsymbol{\pm0.017}$ | **1.000**$\boldsymbol{\pm0.000}$ | **0.475**$\boldsymbol{\pm0.016}$ | **1.000**$\boldsymbol{\pm0.000}$ |
| 40 | 0.493$\pm0.017$ | 0.498$\pm0.019$ | 0.343$\pm0.027$ | 0.347$\pm0.017$ | $0.502\pm0.016$ | 0.493$\pm0.022$ |
| 45 | 0.509$\pm0.020$ | 0.512$\pm0.024$ | 0.349$\pm0.016$ | 0.350$\pm0.016$ | 0.505$\pm0.016$ | 0.509$\pm0.021$ |
| 50 | 0.518$\pm0.018$ | 0.520$\pm0.021$ | 0.360$\pm0.018$ | 0.362$\pm0.018$ | 0.511$\pm0.020$ | 0.514$\pm0.020$ |
| 55 | 0.525$\pm0.017$ | 0.527$\pm0.020$ | 0.370$\pm0.016$ | 0.373$\pm0.017$ | 0.519$\pm0.019$ | 0.522$\pm0.019$ |
| 60 | 0.531$\pm0.018$ | 0.534$\pm0.020$ | 0.381$\pm0.015$ | 0.383$\pm0.016$ | 0.526$\pm0.018$ | 0.528$\pm0.018$ |
| 65 | 0.540$\pm0.018$ | 0.534$\pm0.019$ | 0.392$\pm0.015$ | 0.395$\pm0.016$ | 0.535$\pm0.017$ | 0.537$\pm0.017$ |

**Table S4. Comparison of original and permuted model performance (MCC**$\boldsymbol{\pm}$**SD) across thresholds and planes.** p-values were obtained using Welch’s t-test.

| **Plane** | **Lower thr** | **Upper thr** | **Original MCC** | **Permuted MCC** | **p-value** |
| --- | --- | --- | --- | --- | --- |
| **Axial** | 0.50 | 0.50 | 0.497$\pm0.019$ | 0.010$\pm0.021$ | 3.39$\times{10}^{-21}$ |
|  | 0.45 | 0.55 | 0.522$\pm0.018$ | 0.029$\pm0.055$ | 2.01$\times{10}^{-11}$ |
|  | 0.40 | 0.60 | 0.545$\pm0.022$ | 0.040$\pm0.084$ | 3.82$\times{10}^{-9}$ |
|  | 0.35 | 0.65 | 0.567$\pm0.027$ | -0.041$\pm0.129$ | 5.75$\times{10}^{-8}$ |
|  | 0.30 | 0.70 | 0.592$\pm0.027$ | 0.000$\pm0.000$ | 1.16$\times{10}^{-13}$ |
|  | 0.25 | 0.75 | 0.622$\pm0.031$ | 0.000$\pm0.000$ | 2.99$\times{10}^{-13}$ |
|  | 0.20 | 0.80 | 0.654$\pm0.040$ | 0.000$\pm0.000$ | 1.76$\times{10}^{-12}$ |
|  | 0.15 | 0.85 | 0.694$\pm0.043$ | 0.000$\pm0.000$ | 1.98$\times{10}^{-12}$ |
|  | 0.10 | 0.90 | 0.742$\pm0.037$ | 0.000$\pm0.000$ | 3.13$\times{10}^{-13}$ |
|  | 0.05 | 0.95 | 0.843$\pm0.060$ | 0.000$\pm0.000$ | 7.38$\times{10}^{-12}$ |
| **Coronal** | 0.50 | 0.50 | 0.551$\pm0.017$ | 0.020$\pm0.016$ | 1.08$\times{10}^{-23}$ |
|  | 0.45 | 0.55 | 0.583$\pm0.014$ | 0.012$\pm0.042$ | 2.10$\times{10}^{-13}$ |
|  | 0.40 | 0.60 | 0.614$\pm0.014$ | 0.012$\pm0.005$ | 2.73$\times{10}^{-19}$ |
|  | 0.35 | 0.65 | 0.644$\pm0.012$ | 0.021$\pm0.066$ | 9.49$\times{10}^{-11}$ |
|  | 0.30 | 0.70 | 0.676$\pm0.011$ | 0.000$\pm0.000$ | 1.79$\times{10}^{-17}$ |
|  | 0.25 | 0.75 | 0.708$\pm0.015$ | 0.000$\pm0.000$ | 1.55$\times{10}^{-16}$ |
|  | 0.20 | 0.80 | 0.739$\pm0.018$ | 0.000$\pm0.000$ | 3.83$\times{10}^{-16}$ |
|  | 0.15 | 0.85 | 0.766$\pm0.016$ | 0.000$\pm0.000$ | 1.06$\times{10}^{-16}$ |
|  | 0.10 | 0.90 | 0.832$\pm0.025$ | 0.000$\pm0.000$ | 3.46$\times{10}^{-15}$ |
|  | 0.05 | 0.95 | 0.904$\pm0.032$ | 0.000$\pm0.000$ | 1.42$\times{10}^{-14}$ |
| **Sagittal** | 0.50 | 0.50 | 0.533$\pm0.017$ | -0.003$\pm0.014$ | 1.77$\times{10}^{-23}$ |
|  | 0.45 | 0.55 | 0.561$\pm0.016$ | 0.000$\pm0.000$ | 2.04$\times{10}^{-15}$ |
|  | 0.40 | 0.60 | 0.585$\pm0.014$ | 0.000$\pm0.000$ | 3.38$\times{10}^{-16}$ |
|  | 0.35 | 0.65 | 0.615$\pm0.012$ | 0.000$\pm0.000$ | 5.81$\times{10}^{-17}$ |
|  | 0.30 | 0.70 | 0.648$\pm0.020$ | 0.000$\pm0.000$ | 4.31$\times{10}^{-15}$ |
|  | 0.25 | 0.75 | 0.680$\pm0.022$ | 0.000$\pm0.000$ | 6.46$\times{10}^{-15}$ |
|  | 0.20 | 0.80 | 0.722$\pm0.026$ | 0.000$\pm0.000$ | 1.43$\times{10}^{-14}$ |
|  | 0.15 | 0.85 | 0.773$\pm0.033$ | 0.000$\pm0.000$ | 6.66$\times{10}^{-14}$ |
|  | 0.10 | 0.90 | 0.840$\pm0.042$ | 0.000$\pm0.000$ | 3.44$\times{10}^{-13}$ |
|  | 0.05 | 0.95 | 0.745$\pm0.395$ | 0.000$\pm0.000$ | 2.10$\times{10}^{-4}$ |

**Table S5. Classification performance across axial brain slices.** For each slice, the Matthews Correlation Coefficient (MCC), Precision, and Accuracy are reported as mean ± standard deviation across 10 independent runs. Results are based on a binary sex classification task (male vs. female; N = 2,036) using a decision threshold of 0.5.

| **Slice** | **MCC** | **Precision** | **Accuracy** |
| --- | --- | --- | --- |
| 0 | 0.070±0.014 | 0.570±0.003 | 0.565±0.005 |
| 6 | 0.263±0.010 | 0.658±0.008 | 0.640±0.005 |
| 12 | 0.368±0.015 | 0.712±0.012 | 0.689±0.008 |
| 18 | 0.383±0.016 | 0.733±0.017 | 0.693±0.012 |
| 24 | 0.368±0.025 | 0.745±0.020 | 0.681±0.013 |
| 31 | 0.455±0.017 | 0.776±0.015 | 0.727±0.011 |
| 37 | 0.470±0.020 | 0.799±0.011 | 0.731±0.013 |
| 43 | 0.496±0.022 | 0.808±0.018 | 0.745±0.010 |
| 49 | 0.511±0.018 | 0.800±0.016 | 0.755±0.012 |
| 56 | 0.496±0.030 | 0.812±0.022 | 0.742±0.022 |
| 62 | 0.470±0.031 | 0.806±0.021 | 0.727±0.023 |
| 68 | 0.476±0.020 | 0.794±0.021 | 0.736±0.010 |
| 74 | 0.482±0.020 | 0.793±0.037 | 0.737±0.015 |
| **81** | **0.537±0.017** | **0.787±0.017** | **0.771±0.010** |
| 83 | 0.518±0.027 | 0.771±0.028 | 0.761±0.013 |
| 85 | 0.515±0.023 | 0.777±0.016 | 0.760±0.013 |
| 87 | 0.502±0.028 | 0.782±0.020 | 0.752±0.016 |
| 89 | 0.526±0.024 | 0.793±0.014 | 0.765±0.012 |
| 91 | 0.486±0.026 | 0.794±0.027 | 0.741±0.014 |
| 93 | 0.494±0.027 | 0.797±0.011 | 0.746±0.017 |
| 95 | 0.507±0.016 | 0.788±0.015 | 0.755±0.008 |
| 97 | 0.503±0.023 | 0.816±0.013 | 0.747±0.014 |
| 99 | 0.477±0.028 | 0.794±0.021 | 0.736±0.015 |
| 101 | 0.483±0.035 | 0.793±0.022 | 0.741±0.017 |
| 103 | 0.488±0.019 | 0.792±0.023 | 0.743±0.010 |
| 106 | 0.453±0.022 | 0.809±0.016 | 0.717±0.017 |
| 108 | 0.428±0.041 | 0.771±0.030 | 0.711±0.021 |
| 110 | 0.449±0.018 | 0.791±0.026 | 0.718±0.017 |
| 112 | 0.442±0.022 | 0.777±0.024 | 0.719±0.014 |
| 118 | 0.448±0.019 | 0.787±0.022 | 0.720±0.013 |
| 124 | 0.442±0.021 | 0.770±0.023 | 0.720±0.013 |
| 131 | 0.430±0.021 | 0.760±0.020 | 0.716±0.009 |
| 137 | 0.405±0.017 | 0.756±0.018 | 0.701±0.011 |
| 143 | 0.390±0.019 | 0.747±0.031 | 0.693±0.015 |
| 149 | 0.346±0.023 | 0.726±0.026 | 0.672±0.014 |
| 156 | 0.075±0.021 | 0.586±0.019 | 0.552±0.012 |
| 162 | -0.021±0.012 | 0.550±0.015 | 0.541±0.032 |
| 168 | -0.008±0.010 | 0.556±0.000 | 0.556±0.000 |
| 174 | 0.008±0.016 | 0.645±0.177 | 0.534±0.044 |
| 181 | 0.000±0.000 | 0.390±0.255 | 0.556±0.000 |

**Table S6. Classification performance across coronal brain slices.** For each slice, the Matthews Correlation Coefficient (MCC), Precision, and Accuracy are reported as mean ± standard deviation across 10 independent runs. Results are based on a binary sex classification task (male vs. female; N = 2,036) using a decision threshold of 0.5.

| **Slice** | **MCC** | **Precision** | **Accuracy** |
| --- | --- | --- | --- |
| 0 | 0.003±0.015 | 0.628±0.292 | 0.506±0.055 |
| 7 | -0.017±0.013 | 0.564±0.028 | 0.542±0.032 |
| 14 | -0.018±0.013 | 0.556±0.004 | 0.542±0.027 |
| 22 | 0.234±0.018 | 0.675±0.019 | 0.615±0.009 |
| 29 | 0.333±0.019 | 0.712±0.013 | 0.668±0.012 |
| 37 | 0.325±0.024 | 0.724±0.013 | 0.659±0.017 |
| 44 | 0.359±0.014 | 0.729±0.017 | 0.680±0.007 |
| 52 | 0.448±0.017 | 0.770±0.010 | 0.725±0.011 |
| 59 | 0.398±0.018 | 0.749±0.025 | 0.698±0.009 |
| 67 | 0.505±0.017 | 0.782±0.015 | 0.755±0.010 |
| 74 | 0.541±0.013 | 0.806±0.017 | 0.772±0.006 |
| 82 | 0.441±0.021 | 0.758±0.022 | 0.722±0.010 |
| 89 | 0.499±0.018 | 0.788±0.020 | 0.751±0.009 |
| **97** | **0.583±0.021** | **0.832±0.021** | **0.792±0.010** |
| 99 | 0.549±0.011 | 0.808±0.013 | 0.776±0.006 |
| 101 | 0.572±0.017 | 0.828±0.025 | 0.786±0.010 |
| 104 | 0.564±0.026 | 0.819±0.018 | 0.783±0.013 |
| 106 | 0.554±0.013 | 0.813±0.016 | 0.778±0.006 |
| 108 | 0.553±0.021 | 0.812±0.014 | 0.778±0.011 |
| 110 | 0.556±0.017 | 0.824±0.017 | 0.777±0.010 |
| 112 | 0.549±0.017 | 0.818±0.014 | 0.775±0.010 |
| 114 | 0.547±0.016 | 0.799±0.012 | 0.776±0.008 |
| 116 | 0.549±0.017 | 0.814±0.022 | 0.775±0.010 |
| 119 | 0.539±0.021 | 0.814±0.020 | 0.769±0.012 |
| 127 | 0.525±0.021 | 0.812±0.019 | 0.762±0.011 |
| 134 | 0.504±0.020 | 0.798±0.008 | 0.752±0.011 |
| 142 | 0.480±0.026 | 0.775±0.021 | 0.741±0.017 |
| 149 | 0.412±0.016 | 0.762±0.032 | 0.703±0.011 |
| 157 | 0.405±0.024 | 0.746±0.024 | 0.704±0.011 |
| 164 | 0.407±0.019 | 0.753±0.024 | 0.703±0.014 |
| 172 | 0.401±0.017 | 0.753±0.032 | 0.698±0.015 |
| 179 | 0.379±0.029 | 0.750±0.018 | 0.685±0.020 |
| 187 | 0.340±0.017 | 0.727±0.027 | 0.668±0.006 |
| 194 | 0.224±0.014 | 0.675±0.010 | 0.607±0.011 |
| 202 | -0.027±0.013 | 0.551±0.003 | 0.532±0.006 |
| 209 | 0.006±0.009 | 0.604±0.072 | 0.523±0.051 |
| 217 | 0.006±0.009 | 0.634±0.290 | 0.519±0.053 |

**Table S7. Classification performance across sagittal brain slices.** For each slice, the Matthews Correlation Coefficient (MCC), Precision, and Accuracy are reported as mean ± standard deviation across 10 independent runs. Results are based on a binary sex classification task (male vs. female; N = 2,036) using a decision threshold of 0.5.

| **Slice** | **MCC** | **Precision** | **Accuracy** |
| --- | --- | --- | --- |
| 0 | -0.013±0.007 | 0.389±0.255 | 0.556±0.000 |
| 6 | 0.019±0.006 | 0.606±0.097 | 0.535±0.045 |
| 12 | 0.012±0.011 | 0.557±0.004 | 0.534±0.043 |
| 18 | 0.264±0.015 | 0.674±0.019 | 0.636±0.006 |
| 24 | 0.357±0.019 | 0.741±0.022 | 0.675±0.010 |
| 31 | 0.409±0.024 | 0.776±0.019 | 0.698±0.013 |
| 37 | 0.472±0.022 | 0.787±0.022 | 0.735±0.013 |
| 43 | 0.510±0.018 | 0.799±0.014 | 0.755±0.010 |
| 49 | 0.521±0.014 | 0.801±0.021 | 0.760±0.010 |
| 56 | 0.542±0.027 | 0.814±0.026 | 0.771±0.011 |
| 62 | 0.515±0.019 | 0.814±0.015 | 0.755±0.012 |
| 68 | 0.541±0.017 | 0.824±0.020 | 0.769±0.011 |
| 74 | 0.530±0.017 | 0.803±0.016 | 0.766±0.009 |
| 81 | 0.516±0.018 | 0.810±0.019 | 0.756±0.010 |
| 83 | 0.509±0.018 | 0.808±0.018 | 0.753±0.008 |
| 85 | 0.529±0.022 | 0.842±0.015 | 0.757±0.015 |
| 87 | 0.524±0.013 | 0.817±0.018 | 0.760±0.008 |
| **89** | **0.549±0.013** | **0.829±0.022** | **0.772±0.008** |
| 91 | 0.533±0.019 | 0.834±0.016 | 0.762±0.012 |
| 93 | 0.533±0.021 | 0.832±0.011 | 0.762±0.014 |
| 95 | 0.524±0.021 | 0.821±0.016 | 0.759±0.012 |
| 97 | 0.500±0.021 | 0.801±0.025 | 0.748±0.014 |
| 99 | 0.508±0.017 | 0.827±0.025 | 0.747±0.013 |
| 101 | 0.509±0.020 | 0.825±0.034 | 0.747±0.021 |
| 103 | 0.521±0.018 | 0.808±0.026 | 0.760±0.014 |
| 106 | 0.502±0.027 | 0.816±0.016 | 0.747±0.014 |
| 108 | 0.489±0.020 | 0.811±0.028 | 0.739±0.020 |
| 110 | 0.502±0.035 | 0.816±0.019 | 0.746±0.022 |
| 112 | 0.483±0.018 | 0.784±0.026 | 0.741±0.010 |
| 118 | 0.489±0.036 | 0.776±0.029 | 0.747±0.017 |
| 124 | 0.487±0.014 | 0.788±0.024 | 0.743±0.008 |
| 131 | 0.478±0.013 | 0.784±0.020 | 0.739±0.011 |
| 137 | 0.447±0.022 | 0.771±0.026 | 0.723±0.010 |
| 143 | 0.421±0.022 | 0.761±0.017 | 0.709±0.015 |
| 149 | 0.362±0.021 | 0.754±0.026 | 0.672±0.019 |
| 156 | 0.345±0.017 | 0.714±0.015 | 0.675±0.009 |
| 162 | 0.122±0.026 | 0.603±0.016 | 0.574±0.011 |
| 168 | 0.010±0.010 | 0.557±0.001 | 0.556±0.002 |
| 174 | 0.000±0.012 | 0.570±0.062 | 0.534±0.045 |
| 181 | -0.018±0.018 | 0.256±0.264 | 0.534±0.045 |

**Table S8. Classification performance and brain tissue coverage for whole, hemispheric, and peripheral slices in the axial and coronal planes.** Useful voxel counts represent the mean number of nonzero pixels per slice across samples. MCC values are reported as mean ± standard deviation across 10 repeated runs. Hemispheric slices (Left/Right) were obtained by splitting the optimal slice along the midline. Peripheral slices were selected to match the tissue coverage of the hemispheric inputs while originating from anatomically distinct regions.

| **Plane** | **Useful voxel counts** | **MCC** |
| --- | --- | --- |
| Axial #85 | 19514.68 | 0.596 $\pm$ 0.015 |
| Axial #85 Left | 9922.26 | 0.554$\pm$0.020 |
| Axial #85 Right | 9592.42 | 0.478$\pm0.033$ |
| Axial #37 | 10474.8 | 0.450$\pm0.016$ |
| Axial #131 | 9817.5 | 0.420$\pm0.019$ |
| Axial #124 | 12283.7 | 0.430$\pm0.022$ |
| Coronal #97 | 15032.46 | 0.605 $\pm$ 0.026 |
| Coronal #97 Left | 7677.55 | 0.532$\pm0.027$ |
| Coronal #97 Right | 7354.91 | 0.534$\pm0.013$ |
| Coronal #37 | 6302.6 | 0.337$\pm0.021$ |
| Coronal # 44 | 8774.3 | 0.402$\pm0.014$ |
| Coronal # 164 | 7972.9 | 0.443$\pm0.018$ |
